# Postmortem Protein Degradation as a Tool to Estimate the PMI: A Systematic Review

**DOI:** 10.3390/diagnostics10121014

**Published:** 2020-11-26

**Authors:** Angela Zissler, Walter Stoiber, Peter Steinbacher, Janine Geissenberger, Fabio C. Monticelli, Stefan Pittner

**Affiliations:** 1 Department of Biosciences, University of Salzburg, 5020 Salzburg, Austria; angela.zissler@sbg.ac.at (A.Z.); walter.stoiber@sbg.ac.at (W.S.); peter.steinbacher@sbg.ac.at (P.S.); janine.geissenberger@stud.sbg.ac.at (J.G.); 2 Department of Forensic Medicine, University of Salzburg, 5020 Salzburg, Austria; fabio.monticelli@sbg.ac.at

**Keywords:** protein, degradation, postmortem interval, time since death

## Abstract

**Objectives:** We provide a systematic review of the literature to evaluate the current research status of protein degradation-based postmortem interval (PMI) estimation. Special attention is paid to the applicability of the proposed approaches/methods in forensic routine practice. **Method:** A systematic review of the literature on protein degradation in tissues and organs of animals and humans was conducted. Therefore, we searched the scientific databases Pubmed and Ovid for publications until December 2019. Additional searches were performed in Google Scholar and the reference lists of eligible articles. **Results:** A total of 36 studies were included. This enabled us to consider the degradation pattern of over 130 proteins from 11 different tissues, studied with different methods including well-established and modern approaches. Although comparison between studies is complicated by the heterogeneity of study designs, tissue types, methods, proteins and outcome measurement, there is clear evidence for a high explanatory power of protein degradation analysis in forensic PMI analysis. **Conclusions:** Although only few approaches have yet exceeded a basic research level, the current research status provides strong evidence in favor of the applicability of a protein degradation-based PMI estimation method in routine forensic practice. Further targeted research effort towards specific aims (also addressing influencing factors and exclusion criteria), especially in human tissue will be required to obtain a robust, reliable laboratory protocol, and collect sufficient data to develop accurate multifactorial mathematical decomposition models.

## 1. Introduction

Estimation of time since death represents a central aspect and a complex task in daily forensic casework. In homicides, suicides and unintentional deaths, time since death must be estimated as reliably and precisely as possible, since an erroneous postmortem interval (PMI) impacts the course and outcome of criminal investigations [1]. Besides the testimonies of eyewitnesses and non-scientific criminalistic or scene markers, scientific biomedical techniques are the main sources of time since death estimation [2]. Such PMI delimitation methods rely on biochemical, physical and physicochemical body changes, inevitably occurring after death due to the lack of circulating oxygen, cessation of anabolic production of metabolites, altered enzymatic reactions and cellular degradation [3]. These methods allow forensic scientists to calculate back to the starting point of the body changes (i.e., time of death) along a time-dependent curve.

Commonly applied biomedical methods of PMI determination include the temperature-based method of Henssge, the electrical and mechanical excitability of skeletal muscles, pharmacological excitability of the iris, progress of rigor mortis, and postmortem lividity development [1,4]. In certain cases, morphological changes can aid in determining a minimum PMI [1,5]. These methods, however, are limited to particular postmortem phases, and often cannot be sensibly applied due to specific restrictions (e.g., under specific environmental conditions, or circumstances surrounding death such as injuries). Thus, numerous additional approaches have been proposed in recent years to overcome the deficits and gaps in the applied spectrum of conventional PMI delimitation methods. Many of the new approaches focus on biochemical alterations. Inclusion of biochemical methods in a compound approach can particularly help eliminating examiner bias, which always imposes a risk factor within the conventional methods such as analysis of rigor mortis, livor mortis and putrefaction [6]. Substrates considered susceptible to PMI-related degradation include body fluids such as blood (serum), spinal fluid, vitreous humour, pericardial fluid, and synovial joint [7,8,9], as well as molecular constituents (biomarkers) of various body tissues.

In recent years, proteins in particular have been evaluated for their potential to aid PMI delimitation. Serum levels of blood-derived marker proteins (e.g., insulin, immunoglobulin M, creatine kinase, etc.) were monitored over different PMI [10,11,12], and postmortem protein degradation in different tissues was followed over time. The latter in particular has turned out to be a promising approach. The breakdown of proteins within tissues is a ubiquitous and evolutionary conserved process, with main mechanisms being also exerted in living body cells [13]. Thus, hydrolysis of peptide bonds can either be catalyzed by proteolytic enzymes (proteases) or non-enzymatically, as for example under extreme pH and temperature conditions [14]. In protein turnover of living tissues, proteolysis is mediated enzymatically by proteases via different mechanisms [15]. The ATP-independent calpain system is usually inhibited by calpastatin and low cytoplasmic calcium concentrations [16]. After death, however, calpains are extensively activated due to the postmortal increase of intracellular calcium [15,17], and are thought to be responsible for a large part of postmortem proteolysis [18,19]. Additionally, postmortem protein decomposition is accelerated by microbial enzymes, and in later phases possibly as well by enzymes released from necrophagous insects [20].

Recent evidence has demonstrated that postmortem degradation of certain proteins proceeds in a regular predictable fashion. Therefore, several groups have suggested analysis of the time course of protein degradation as a promising tool in forensic PMI estimation [21,22,23]. To evaluate the current research status and progression of protein degradation-based PMI estimation, the present review systematically identifies and summarizes studies that harnessed proteins from animal and/or human tissues for this purpose. This is combined with and supplemented by an assessment and discussion of study quality (respectively the risk of bias for meta-analysis) in order to clarify the body of evidence regarding tissue, marker proteins and methodology currently available for use in forensic PMI estimation.

## 2. Methods

We conducted an evidence based systematic review of the literature according to the PRISMA (Preferred Reporting Items for Systematic Reviews and Meta-Analysis) guidelines to evaluate the current research status and progression of protein degradation based PMI estimation. A focused research question was defined using a PICo (Population, Interest, Context) framework, serving to assess whether analysis of protein degradation is an effective method to estimate the PMI (I = Interest) in postmortem tissues and organs of animals and humans (P = Population/Problem/Condition) for the purposes of forensic science (Co = Context/Setting).

### 2.1. Data Sources and Eligibility Criteria

A thorough search of the literature was performed using the electronic databases PubMed, Ovid, Google Scholar, and the reference lists of articles published on the subject. Studies were preselected based on the following inclusion criteria: peer revision, publishing date between January 1968 and 31 December 2019, English language, and full text availability.

### 2.2. Search Strategy

Using different Boolean operators, the systematic search was conducted as follows: the terms (postmortem OR post mortem OR post-mortem) AND (protein degradation OR protein decomposition) were consecutively combined with keywords and related terms describing the tissues/organs of interest including heart muscle, skeletal muscle, lung, brain, kidney, liver, pancreas, spleen, skin, bone, cartilage, and teeth: 1. AND (myocard OR heart OR muscle); 2. AND (lung OR pulmonary OR respiratory); 3. AND (brain OR neural); 4. AND (kidney OR nephrotic); 5. (liver OR hepatic); 6. (pancreas OR pancreatic); 7. AND (spleen); 8. (skin OR dermal); 9. (bone OR skeletal OR skeleton); 10. (cartilage OR chondral); 11. (teeth OR pulp). Upon completion of this evaluation, a hand search for references cited in the identified studies was performed. In addition, an extended Google Scholar search to also identify studies not listed in Pubmed and Ovid (e.g., studies published in open access journals) was undertaken to identify further related articles using the keywords for selected tissues and/or organs as described above, variously combined with the terms ‘time since death’ and ‘forensic’ and ‘postmortem interval’. The last search date was 31 December 2019.

### 2.3. Study Selection

After identification of the records through database searching, duplicates were removed. Two reviewers independently screened the remaining articles based on the evaluation of titles and abstracts. The following data were recorded: study content (e.g., meat science, forensic sciences, etc.), type of article (e.g., research article, review, etc.), type of research target model (tissue/organ or cell culture), and type of tissue/organ in case of animal studies and studies on tissues of human corpses. Based on this evaluation, articles with irrelevant content (other than forensic) were excluded from further evaluation. Original research articles, reviews (including meta-analyzes), and case studies were included; all other article types were excluded. Articles using cell cultures as primary research model, and articles that did not analyze the degradation of a specific tissue and/or organ (e.g., entomology studies, microbiome studies) were also excluded. All articles of relevant form, i.e., those indicating evaluation of animal and/or human tissues in a forensically relevant context, were included and screened for eligibility. Discrepancies between the two reviewers regarding eligibility were discussed until a consensus was reached.

The full-text versions of the remaining studies were evaluated to determine final eligibility. Only studies with the specific aim to investigate postmortal changes during the PMI in a forensic setting using proteins as selected biomarkers were retained in the final sample. Work evaluating other forensic issues (e.g., wound age estimation, cause of death, etc.) and articles using biomarkers such as DNA and RNA were excluded.

### 2.4. Risk of Bias Assessment

To our knowledge, there is currently no published risk of bias assessment tool that could be applied to the study types analyzed in this review. However, as we use ‘study quality’ and ‘potential bias’ as important criteria to estimate and rate the significance and reliability of study outcomes, we generated a model framework to estimate the overall quality and risk of bias of the included studies.

To assess the internal validity of the articles, two of the authors formulated specific signaling questions based on the Cochrane Risk of Bias tool RoB 2.0 [24]. This yielded in a risk of bias and quality assessment framework suitable for application to the finally included studies (for detailed information refer to supplementary material Files S1 and S2). These signaling questions refer to 6 domains including general study design, precision in reporting, presence of original outcome data, outcome measurements, selective outcome reporting, and multiple use of data.

Assessment of risk of bias and methodological quality of each included study was performed independently by four of the review authors. Disagreements were resolved by discussion between the authors. Items were scored as having low risk of bias, or moderate risk of bias, or high risk of bias and, referring to the recommendations of the Cochrane Risk of Bias collaboration [24]. The review of Li et al. [25] was omitted from the trial due to different study type and insufficient information regarding the included studies.

### 2.5. Data Extraction and Synthesis

Due to the heterogeneity of the identified studies in relation to experimental design and concomitant fundamental differences in outcome measurements and results, data could not be collapsed for meta-analysis, but were instead synthesized in a descriptive approach. For this purpose, study characteristics were extracted independently by two of the authors in consultation with all authors using standardized data extraction forms. The following data were extracted: (1) general characteristics of design and aim of the study including year of publication, study type, number of individuals and sample size, study groups, type(s) of tissues and model organism(s), as well as type(s) of method(s) used, (2) detailed information regarding target species, PMI, sampling location and frequency, storage conditions, and investigated proteins, and (3) type of outcome and main study outcome. Extracted data were cross checked for accuracy.

Studies were compared in terms of study design, outcome and the obtained risk of bias of the individual studies. Appraisal of evidence across the studies was carried out in relation to methods of investigation, type of tissue and type of proteins. The ‘NHMRC (National Health and Medical Research Council) approach to grade evidence recommendations’ [26] served as a template for developing a strength of the body of evidence matrix for the non-clinical studies included in this review. The authors therefore adapted the NHMRC approach to take into account the evidence base (number of studies, level of evidence and study quality in form of risk of bias), the consistency of findings, and the generalizability of the results on behalf of predefined measures. For details see supplementary material (File S3).

The authors disclose that utmost consideration was paid to a most objective assessment of risk of bias and strength of the body of evidence. Nevertheless, some aspects had to be extensively discussed to reach a consensus. The outcome of this systematic review is presented below, according to our best scientific practice.

## 3. Results

### 3.1. Study Selection

**Figure 1:**
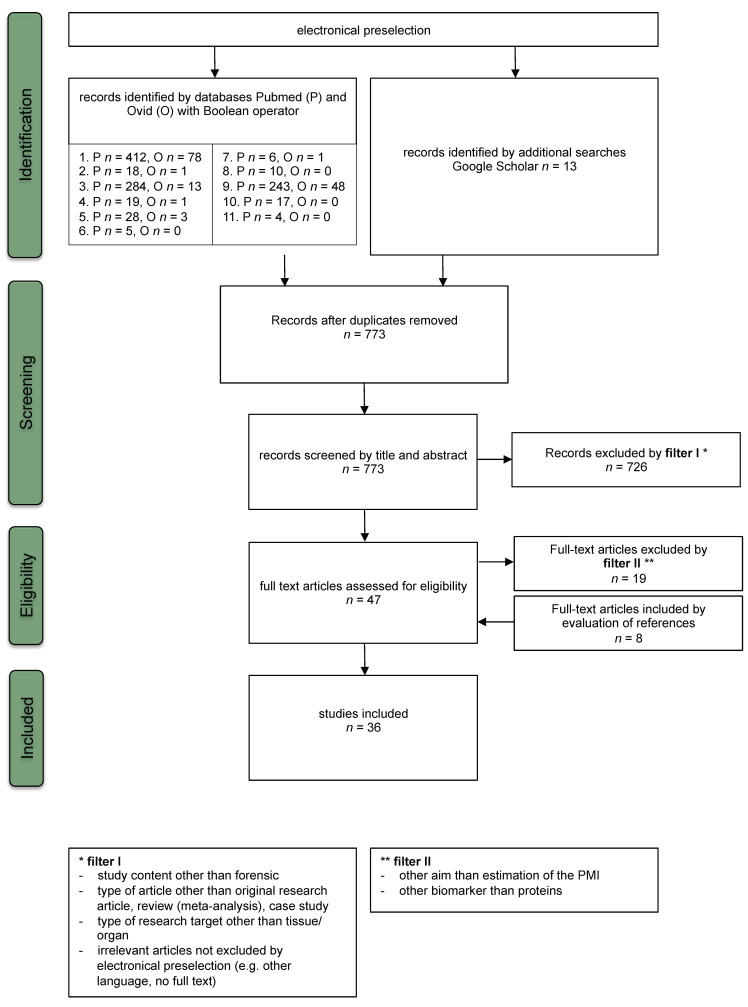
Flow chart of the literature search process and study selection according to PRISMA (Preferred Reporting Items for Systematic Reviews and Meta-Analysis) guidelines. To generate Boolean operators for searches in PubMed (P) and Ovid (O), the following phrases were combined with “AND (postmortem OR post mortem OR post-mortem) AND (protein degradation OR protein decomposition)”. 1: “(myocard OR heart OR muscle)”, 2. “(lung OR pulmonary OR respiratory)”, 3. “(brain OR neural)”, 4. “(kidney OR nephrotic)”, 5. “(liver OR hepatic)”, 6. “(pancreas OR pancreatic)”, 7. “(spleen)”, 8. “(skin OR dermal)”, 9. “(bone OR skeletal OR skeleton)”, 10. “(cartilage OR chondral)”, 11. “(teeth OR pulp)”.

The systematic search in the Pubmed and Ovid platforms yielded a total of 1191 records (Figure 1). The expanded searches in Google Scholar resulted in different numbers of hits, depending upon the combination of search terms used. Using the keyword combinations ‘cardiac AND time since death’, ‘cartilage AND forensic’, ‘liver AND postmortem interval’, ‘muscle AND postmortem interval’, and ‘pancreatic AND time since death’, these searches rendered another thirteen studies considered to contain relevant content. A total of 1204 studies were then searched for duplicates. After duplicate removal (*n* = 431), a remaining number of 773 studies was evaluated by screening article titles and abstracts. 726 of these articles were found not to meet the inclusion criteria of the first filter. Most of the studies omitted by this step had content other than forensic science, dealing with meat science and clinical matters instead. Of the 47 remaining studies, 19 were excluded because their aim was not to evaluate postmortal changes during the PMI and/or PMI estimation (rather dealing with matters relating to cause of death, individual age, wound age, and clinical or veterinary research), and/or because they used biomarkers other than proteins (e.g., RNA, DNA, chemical elements). Finally, screening of the references of the remaining 28 full text articles enabled to identify 8 further valid studies that met all relevant inclusion criteria. Thus, a total of 36 studies were found appropriate to be included in this review.

### 3.2. Study Characteristics

All included studies aimed to improve the current knowledge on postmortem protein degradation to delimitate the PMI. Studies investigated either samples of human origin, or samples from animal models, or both (Table 1). Sample sizes covered a broad range, from *n* = 2 to *n* = 500 (Table 2). Human studies analyzed tissues collected from autopsy cases with (for the most part) known PMI. In some of these studies, the tissue of interest was stored over a certain period of time and sampled at several postmortem time points. Sampling at sequential time points was also carried out in most of the animal studies. Four studies investigated protein degradation under different thermal storage conditions; 34 of the 36 studies included are research articles evaluating protein (or proteome) degradation in early, intermediate and late PMI. One of the two remaining studies is a case study applying a protein-based PMI estimation method to trace the progression of events in a crime. The second (Li et al. [25]) is a review paper summarizing the results of 6 original research articles in the Chinese language.

**Figure 2:**
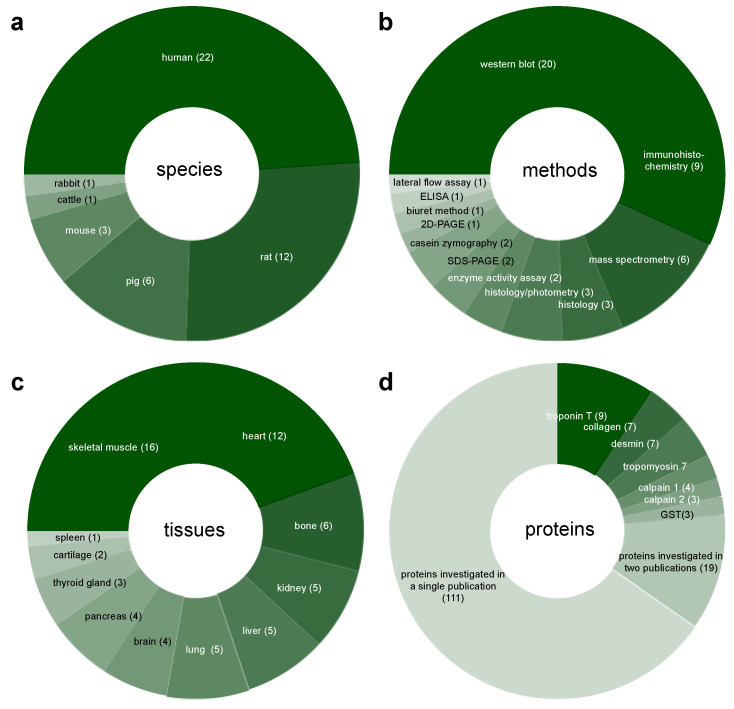
Frequency diagrams of analyzed species (**a**), methods (**b**), tissues (**c**), and proteins (**d**) targeted in at least two studies. All investigated proteins are listed in Table S1.

If classified only by origin of tissue, a total of 22 articles (20 research articles [23,27,28,29,30,31,32,33,34,35,36,37,38,39,40,41,42,43,44,45], and 2 of the studies reported on in the review of Li et al. [25]) investigated samples from human cases; 23 articles worked on samples from animal models. Animal species utilized include rat [25,42,45,46,47,48,49,50,51,52], pig [21,53,54,55,56,57], mouse [22,45,58], cattle [33] and rabbit [25] (Figure 2a, Table 2).

The spectrum of methods employed to analyze protein decomposition in forensic PMI research is broad (12 different methods) (Table 1, Figure 2b). A majority of the studies (16 research articles [21,22,33,34,35,36,37,38,39,45,46,48,49,52,54,55] and 4 of the studies in the review of Li et al. [25]) used Western blotting. Immunohistochemistry (IHC) was used in 8 research articles [27,28,29,30,31,41,48,52]. One further study referred to in Li et al. [25] relied on immunohistochemistry. Proteomic analysis with mass spectrometry (MS) was used in a total of 6 studies [23,40,42,45,51,59]. Types of MS used were: matrix assisted laser desorption/ionization-time of flight MS (MALDI-TOF-MS), liquid chromatography MS/MS (LC-MS/MS), and high-performance liquid chromatography-MS/MS (HPLC-MS/MS). Three studies performed photometry-based assessment of protein concentrations after conventional histological staining and destaining of sections [43,53,56]. In another 3 studies, histological staining was evaluated by digital analysis [43,56] or with the help of a grading scale [44]. Two studies each used sodium dodecyl sulfate polyacrylamide gel electrophoresis (SDS-PAGE) [21,55], enzyme activity assays (with subsequent photometrical analysis) [47,58], and casein zymography [21,38]. Further methods included two-dimensional polyacrylamide gel electrophoresis (2D-PAGE) [50], enzyme-linked immunosorbent assay (ELISA) [32], lateral flow assay (LFA)–based chip [48], and the Biuret method to calculate the myofibril fragmentation index [25].

Categorized by source of tissue, the original studies included in this review examined tissue samples from 10 different sources (Table 1, Figure 2c). These included internal organs (brain, heart, kidney, liver, lung, pancreas, thyroid gland) and tissues of the musculoskeletal system (skeletal muscle, bone, cartilage). The review article [25] additionally reports on the spleen. Broken down by frequency of use, the most frequently analyzed tissue was skeletal muscle. It was used in 11 research articles [21,22,38,39,45,46,48,49,51,55,58] and the one case study [39], as well as in 5 studies described in the review article of Li et al. [25]. Cardiac muscle tissue was used in 7 of the research studies [32,33,34,35,36,37,50], and in 4 studies of the review article [25]; 6 research articles used bone [40,43,53,56,57,59], 4 research articles relied on the liver [32,42,50,58], another 4 on the lung [22,25,46,52], as did one article of the review [25]). Brain served as target tissue in 3 research articles [27,32,58] and in 1 article in the review [25], the kidney in 3 research articles [32,48,58] and again in 1 article in the review [25], and the pancreas in 4 original research articles [27,28,29,41]). The thyroid gland [30,31,41] was used 3 times, cartilage 2 times [44,54], and the spleen one time [25].

Disregarding proteomic studies that do not address individual proteins, the articles included in this review report on PMI-related degradation patterns of over 130 specific proteins (including subtypes, isoforms, and subunits). All protein names mentioned are adopted from the respective articles and are listed in the supplemental materials (Table S1). Proteins and their subtypes, isoforms and subunits investigated in two or more studies are represented in Figure 2d, and further addressed below and in the discussion. The most frequently analyzed protein was the structural protein troponin T (used in 9 studies [21,34,35,36,37,38,39,50,55]), followed by desmin and tropomyosin (7 articles each, including the case study [21,38,39,45,49,50,55]). Collagen proteins were also analyzed in 7 articles [23,40,43,44,53,56,57], the study of Prieto-Bonete et al. [23] notably including 14 subtypes/isoforms. The activity/postmortem degradation of the enzyme calpain 1 (µ-calpain) was investigated in 3 of the research articles and in the case study [21,38,39,55]. Similarly, calpain 2 (m-calpain) [21,38,39] and glutathione-S-transferase [47,50,58] were also targeted in 3 articles each. Kwak et al. [50] investigated two isoforms of glutathione-S-transferase. Nineteen further proteins were also used in 2 studies each, and all other proteins (as listed in Table S1) were tested only singly.

### 3.3. Risk of Bias Assessment

**Figure 3:**
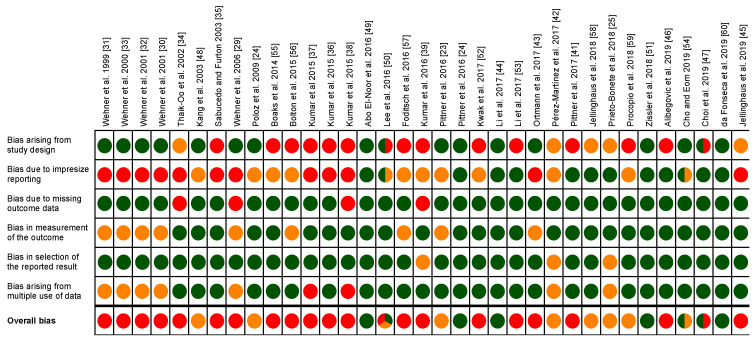
Risk of bias assessment of included studies. Green = low risk of bias, yellow = moderate risk of bias, red = high risk of bias. Mixed colors indicate that parts of the study were assessed with different risks of bias.

Details concerning the risk of bias assessment of included studies are presented in Figure 3 and in Table S2. Due to incomplete information about the included original articles, no risk of bias assessment could be performed for the review article of Li et al. [25]. A large part of the studies (21) were rated with high risk of bias. This is particularly due to the fact that many studies have a pilot character and use only small sample sizes (e.g., one sample per time point). Seven studies were associated with a moderate risk of bias, and 8 studies with a low risk of bias (note that three papers were multi-rated, see explanation in Table S2). In terms of the year of publication, study quality has generally tended to improve in recent years. The studies of Wehner, Ortmann, Pittner, Pérez-Martínez and Prieto-Bonete, and respective co-workers [23,27,28,29,30,31,38,40,41], used large sample sizes (up to 500 corpses), samples being in most cases delimited by predefined exclusion criteria, and presented individual data of corpses and influencing factors. All human studies included only cases with known (approximate) PMI. The animal studies used a mean of 24 cadavers per study, ranging from 2 to 84 animals. When using smaller animals (mice and rats), sample sizes were larger compared to work on larger animals (pigs). With pigs in particular, some studies investigated separated body parts (e.g., legs, bones) to increase sample size. Most of the analyzed animal studies used zero-hour samples for reference, and samples from various postmortem time points. Detailed descriptions of study designs and clear outcome reporting is available in most of the studies, also with a trend to improve in recent years. Usually, influencing factors were also reported. By contrast, presentation of sampling procedure often lack details, and imprecise reporting about applied measurement procedures and data analyzes are common deficits, together resulting in poor reproducibility. For example, only one study [43] reports about blinding of the assessors. Especially a combination of subjective evaluation and lack of blinding can often increase the risk of bias. This applies in particular to the studies of Wehner et al., Ortmann et al., Foditsch et al., and Pittner et al. [21,27,28,29,30,31,41,55], which all do not sufficiently address how intensities (e.g., of immuo-staining, or Western blot bands) were rated. For some studies, it appears that individual corpses were sampled for several analyzes and published in separate manuscripts. Basically, this does not worsen the quality of the individual studies, but can/is likely to influence meta-analyses. Possible bias from this source exists especially for the publications of Kumar et al., as some resulting data seem to be used several times in different papers (e.g., [35,36]).

### 3.4. Protein Degradation as a Tool for Postmortem Interval (PMI) Estimation

**Figure 4:**
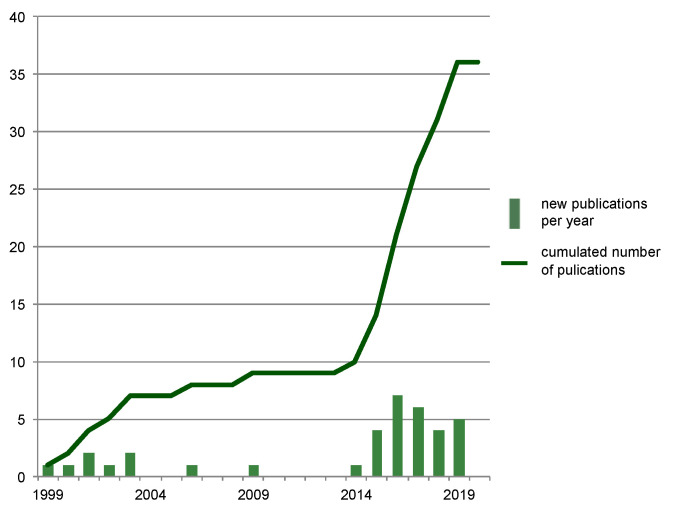
Timeline of published studies. The first included study aiming to determine time since death by means of protein degradation analysis was published in 1999. At around 2015, the number of published articles in this field increased significantly and remained on an elevated level up to present.

The first research efforts addressing the correlation of postmortem protein degradation and PMI arose in the late 1990s and early 2000s (Figure 4). It took until around 2015 for numbers of published articles in this forensic field to increase significantly, but then they remained at an elevated level up to present, illustrating the lasting interest in the topic and demand for further research.

A summary overview of study characteristics (type of protein and tissue of origin, donor species, method of analysis, number of samples, outcome details) is provided in Table 2. A detailed summary with a comment on all included studies also making allowance for historical context is provided in supplementary results (File S4).

## 4. Discussion

The present systematic search of the relevant literature discloses that over the last 20 years, a remarkably broad spectrum of molecular methods has been tested for their suitability to improve forensic PMI estimation. The amount of valid literature is rather heterogeneous in both topic (regarding the specific approach) and scientific quality. This partly impedes comparison of results across studies. Nevertheless, the overall evidence demonstrates clearly that analysis of protein degradation is a highly promising tool to determine the PMI.

Among the 36 studies that passed the selection filters for systematic revision, a large part have a pilot character with a preliminary research level, often entailing assignment of a high risk of bias. Only a few investigations progressed to a more advanced level, with large-scale experimentation on human tissue. Altogether, more than 130 proteins from 11 different tissues of human and animal origin were analyzed with both well-established and novel methodological approaches. The most commonly assessed outcome parameters were protein concentrations and distinct degradation events (i.e., presence/absence of proteins and their fragments) in different postmortem time frames. Overall scientific quality and conclusive reporting were found to have improved in recent years. Specific aspects of the studies are discussed in more detail below.

### 4.1. Body of Evidence

#### 4.1.1. Evidence Base and Consistency

**Figure 5:**
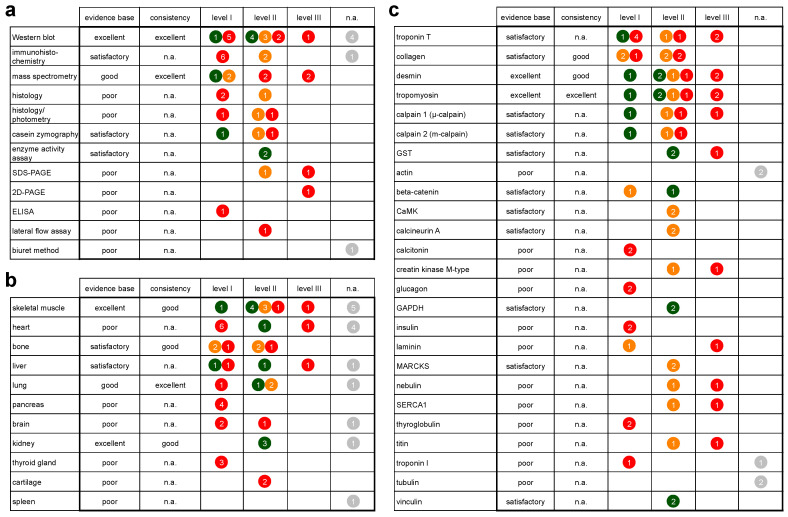
Overview of the body of evidence. Evidence base, consistency and level of evidence are depicted for methods (**a**), tissues (**b**), and proteins (**c**). Studies with low risk of bias are marked in green, those with moderate risk of bias in orange, and those with high risk of bias studies in red. Numbers indicate the amount of corresponding studies. Evidence levels: level I, quantitative studies on human tissue; level II, quantitative animal or human case studies; level III, animal case studies; n.a.: no level applicable (grey) (only referring to studies in review article [25]).

Methods: There is an excellent evidence base for the use of Western blots, and a good evidence base for mass spectrometry (Figure 5a, Table S3 and File S3). Regarding IHC, most studies were associated with high risk of bias, often due to a subjective interpretation and quantification of staining intensity, grading the evidence base for IHC as satisfactory. A similarly satisfactory evidence base exists for the use of enzyme activity assays and casein zymography. Evidence for other methods was graded poor (Figure 5).

Regarding the adequacy of methods to produce consistent outcome, Western blotting and mass spectrometry were the only techniques that passed the criteria to assess consistency (as determined by number of studies with low and moderate risk of bias (for details see Table S3 and File S3, Figure 5). It has to be kept in mind that the probability of inconsistent outcome increases with the number of studies. Therefore, it is remarkable that outcomes of Western blot studies (*n* = 16) are almost entirely consistent, resulting in an excellent rating of the method in this respect. For the vast majority of tested proteins, a significant intensity decrease and/or a complete loss of bands was detected over the investigated PMI. Only some proteins remained stable, and one (protein phosphatase 2A) exhibited an initial rise in intensity under cold conditions, but a significant decrease at room temperature [22]. Consistent outcomes rated excellent have also been asserted for mass spectrometry. Similar to Western blotting, studies mainly found a time-dependent decrease in concentrations, incidentally followed by full loss of the protein, and in some cases also stable presence over a long PMI.

Tissues: In regard to tissues tested for suitability as protein sources, there is an excellent evidence base for skeletal muscle and kidney, a good evidence base for lung tissue, and a satisfactory evidence base for bone and liver (Figure 5). Evidence of other tissues was graded poor (Figure 5). Notably, all tissues except for spleen were also studied in humans. Only skeletal muscle and liver were investigated in low risk of bias studies using human tissue.

Regarding the suitability of organs/tissues to serve as protein donors for PMI estimation, skeletal muscle, bone, lung and kidney passed the respective criteria to assess consistency (Table S3, File S3). The vast majority of the proteins extracted from these organs/tissues was found to degrade in a consistent, significant manner with progressing PMI, while some exhibited a similarly consistent stability. Consistency of findings in lung tissue is graded excellent, consistency of outcome for skeletal muscle, bone and kidney are graded good (Figure 5). It has to be noted that results from lung and kidney exclusively derive from animal models; and skeletal muscle represents the only tissue of human origin investigated in a low risk of bias study so far (Figure 5).

Proteins: There is an excellent evidence base for desmin and tropomyosin, and a satisfactory evidence base for troponin T, collagen, calpain 1 and 2, glutathione-S-transferase, beta-catenin, Ca2+/calmodulin-dependent protein kinase II, calcineurin A, glycerinaldehyd-3-phosphat-dehydrogenase, myristoylated alanine-rich C-kinase substrate and vinculin (Figure 5). Among those proteins, degradation patterns of desmin, tropomyosin, troponin T, collagen, calpain 1 and 2 and beta-catenin were yet tested in humans. Evidence for other proteins was graded poor (Figure 5).

Proteins passing the predefined criteria for consistency assessment were collagen, desmin and tropomyosin (Table S3, File S3). Collagen was rated as having good outcome consistency (Figure 5). This mainly because all relevant studies found collagen to be comparably stable, thus proving its suitability especially as a late PMI marker that can be investigated in detail in bones. Desmin from skeletal muscle was similarly rated with good outcome consistency, with degradation patterns being largely consistent across investigated species (human, pig, rat). Native desmin is lost at intermediate PMIs; in small animals earlier than in large animals. Degradation products with similar molecular weights appeared in comparable time frames across species, again earlier in small animals, later in humans and large animals. In skeletal muscle, tropomyosin presented an excellent outcome consistency, as it remained generally stable over the investigated PMIs in humans and in animals.

#### 4.1.2. Generalizability

Overall, there is strong evidence that protein degradation kinetics (e.g., appearance of certain degradation products) are similar across various mammalian species including humans. However, as to forensic routine case work, it is the generalizability of evidence from human samples that is of interest. Therefore, this aspect was separately assessed for studies reporting on the decomposition of proteins from human tissues. In most of these studies, populations studied represented cases with characteristics, typical for routine forensic practice. The evidence from these studies can therefore directly be judged as generalizable for the target population. However, some caveats remain relating to the impact of the cause of death. Caution is advised in relation to three studies that report on protein degradation in burn and electrocution cases. Here, additional research on the physicochemical details is required to assess the generalizability of the study outcome [34,35,36] (File S3).

### 4.2. Methods

#### 4.2.1. Sample Size

Most the studies used a sufficient sample size with respect to the study aims. Notably, the studies were often directly aimed to detect new PMI markers, or to adapt a method to analyze specific protein-related alterations. As always in quantitative work, calculation of the sample size based on power analyzes is a pivotal aspect also in this field, and should be undertaken whenever possible.

#### 4.2.2. The Use of Control Samples

The selection of representative control samples is one of the most crucial aspects in the context of forensic PMI estimation, and at the same time often the most difficult to manage. Included studies assessed protein abundances at a certain point of time, or at multiple time points postmortem. In animal studies these values were compared to those of “zero-hour” controls. In humans, appropriate controls came from comparisons of “early” versus “late” PMI samples, or from multiple measurements of a large population. However, projections into periods outside the sampled PMI range (earlier than the shortest and/or after the longest PMI) are inadmissible.

#### 4.2.3. Animal Models versus Human Corpses

There is evidence from the results of the analyzed studies that postmortem protein degradation patterns are principally similar across species, which generally supports the use of animal models in protein degradation-based PMI research. Indeed, animal studies have shown that they can supply this research with a solid foundation [21,60], and have contributed to improve medico-legal practice [39]. Well-considered animal models are best suited for the detection of new markers, the analysis of specific influencing factors, and methodological proof of principle. However, animal models enable only limited progress for forensic routine application. There are no studies available comparing different species with similar study settings, but protein decomposition rates in humans in particular may be more pronounced in terms of their difference from those of animal models (especially of small model organisms such as rodents) [61]. This partly relativizes results from animals as data cannot be directly transferred. Thus, there is no way around investigating human decomposition in this regard, which inevitably relates back to the important requirements of appropriate sample sizes and multiple sampling of individuals. Considering this, especially anthropological research facilities may offer the conditions to conduct realistic semi-controlled longitudinal research using samples from donated human remains with well-known PMI [62].

#### 4.2.4. Protein Identification

Among the top three methods tested in the forensic context, immunohistochemistry and Western blotting used antibodies to establish whether predefined proteins are susceptible to time-dependent proteolysis, while mass spectrometry was mostly used to profile the whole proteome, identifying changes in the presence of proteins/peptides retrospectively. Over the time period covered by this review, the identification of proteins was generally facilitated by improved availability of specific antibodies and databases for best match identification.

#### 4.2.5. Data Assessment and Analysis

Data were assessed either by qualitative observations and/or in a (semi)-quantitative manner. Additionally, two main approaches of outcome acquisition and data analysis have been followed: (i) calculation and comparison of gradual alterations of protein concentration levels, and (ii) description and comparison of protein presence (or absence) at certain time points postmortem. Especially when analyzing gradual changes, the validity of the approach depends considerably on the availability of generally applicable starting values [39]. These values can be feasibly established with standardized experiments in animal models (“zero-hour samples”), but it is challenging to achieve the same with human samples, and generally impossible in forensic cases, where antemortem reference or at-death-data are usually lacking [1,39]. A different approach circumventing known starting values is to use reference *(*“housekeeping”) proteins for normalization [52], which is a commonly followed strategy in protein expression analysis. This, however, cannot be generally recommended for research on postmortem tissue as also housekeeping proteins are in all probability likewise susceptible to proteolysis. This is best exemplified by the postmortem degradation of glyeraldehyde-3-phosphate dehydrogenase (GAPDH) [45,48] and alpha-tubulin [63], both commonly used for normalization [64].

Thus, with the current state of research, the preferred future strategy for developing PMI estimation methods should be to rely on strict detection limits (protein present/absent) based on rigorous concentration thresholds, avoiding qualitative subjective assessment to reduce risk of bias.

#### 4.2.6. Applicability of Methods for Forensic Case Work and Future Investigation

None of the approaches tested is as yet broadly applied in forensic routine. Nevertheless, there is good evidence indicating a broader future applicability of protein degradation analysis by Western blotting. This is especially supported, as (i) a majority (16) of the studies use Western blots as preferred method and 8 of which could be associated with low or moderate risk of bias level 1 and 2 (Table S3, File S3); (ii) by the fact that numerous laboratories around the world already have methodical expertise for practical application of the method; and (iii) the technique has already been tested for applicability in a forensic case investigation (under very specific circumstances) [39].

Also the capability to produce results regarding time since death directly at the crime scene can be a decisive factor for a method’s routine applicability. In this direction, the lateral flow assay introduced by Lee et al. [48] may represent a valuable basis. Although as yet only a pilot approach, it seems worthwhile to undertake the development work necessary to establish a routinely applicable, easy-to-use device utilizing this technique.

### 4.3. Tissues

#### 4.3.1. Rate of Tissue Decomposition

**Figure 6:**
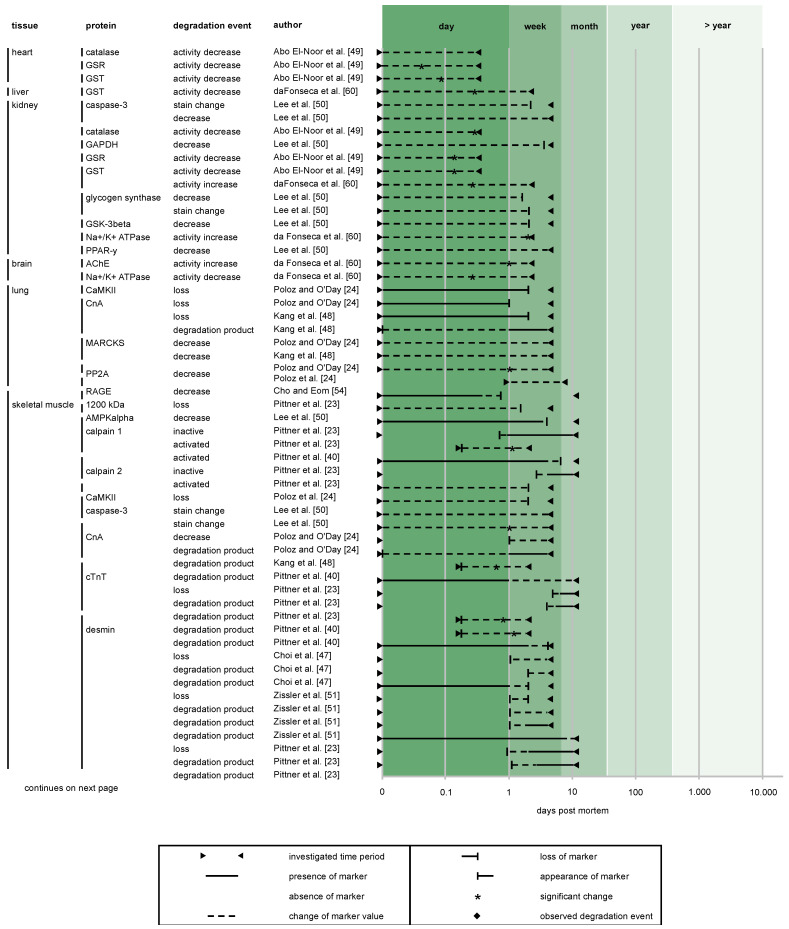

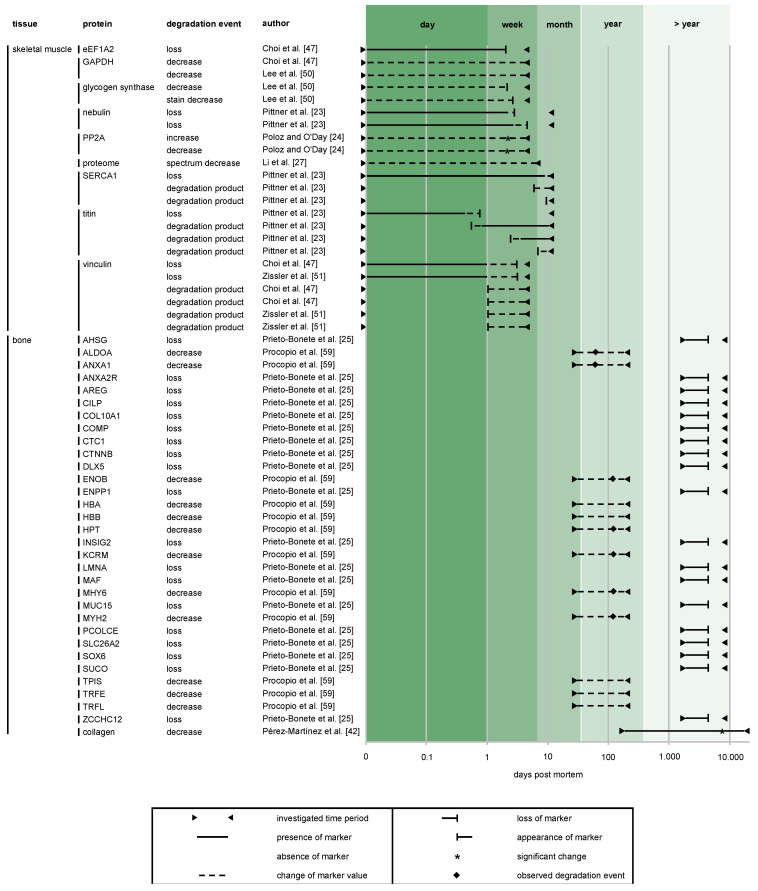
Summary of postmortem degradation events of proteins in various tissues over investigated PMI in logarithmic scale, demonstrating the applicability of tissues and proteins in respective postmortem time frames. The figure includes all proteins originating from studies assigned with a low and moderate risk of bias. Temporal references (day, week, month, year, >year) are indicated by different background colors.

It is known that several characteristics accelerate the rate of tissue decomposition. These include (i) high enzymatic activity, (ii) exposition to a distinct bacterial flora, and (iii) low content of collagen and keratin (“soft tissue” versus “hard tissue”) [65,66,67]. Also, external influences such as accessibility to insects, environmental conditions etc. have to be taken into account. Thus, consideration of these differences is reasonable when defining specific target tissues for protein-based PMI estimation. Indeed, research on protein degradation to date has demonstrated that specific tissues have specific decomposition dynamics, for example enabling the use of cartilage and bone for investigation of the late PMI, periods ranging from months to years (Figure 6). Obviously, soft tissues, by contrast, proved suitable for analyzes in early to intermediate PMIs, ranging from hours to days. However, as opposed to general histological decay the susceptibility of specific proteins to proteolysis seems to be a key determinant for choosing target tissues for a distinct PMI range. This is exemplified by pancreatic tissue. As an enzyme-rich internal organ, it is among the first to be affected by postmortem autolysis [68] (histological analysis show advanced autolytic changes already at 24 hpm, and complete autolysis of Langerhans islets at 36 hpm, when human corpses are stored at room temperature [69]). However, Wehner et al. [29] found that staining of insulin as a PMI delimitation marker is possible until up to 29 dpm, even in advanced autolytic and putrefactive changes.

Four of the studies included in this review investigated the usability of different tissues for PMI estimation with similar study settings. Kang et al. [46] and Poloz and O’Day [22] analyzed lung and skeletal muscle of rat and mouse, respectively. Lee et al. [48] compared protein degradation of rat kidney and skeletal muscle, and da Fonseca [58] tested the activation of specific enzymes in mouse liver, brain, skeletal muscle and kidney. In mice, degradation of calcineurin (Cn) A was found to be faster in lung compared to skeletal muscle [22,46] (Figure 6), but no similar tissue-dependent differences regarding protein degradation susceptibility were found for other proteins so far. Similar investigations in humans are required, as temporal sequences of protein decay in human bodies are in all probability different to those of animals.

#### 4.3.2. Sampling Site

Several of the included studies did not report the precise location of tissue sampling within organs, or other details of the sampling process, resulting in a high risk of bias appraisal. Absence of such information impedes meta-analysis of study data, because no allowance is made for uneven postmortem decomposition due to in-homogeneity within the organ. As a consequence, samples dissected from different organ regions may exhibit different protein degradation patterns as indicated for bone tissue. In long bones, the loss of collagen was found to be accelerated in endosteal and/or periosteal regions compared to mesosteal regions [53].

The as yet underinvestigated influence of topology becomes even more relevant if considering entire organ systems such as the musculoskeletal system which potentially offers a variety of target tissues (muscle, cartilage, bone) and sampling sites distributed over the entire body. This is well depicted in the studies analyzed here, as different muscles (M. biceps femoris [21,55], M. vastus lateralis [38,39,45], M. quadriceps femoris [49,51], M. gastrocnemius [58], and M. psoas [48]), different long bones (tibia [40,53,57], humerus [40,53] and femur [23,53]) and different cartilage sites (metacarpal/metatarsal-phalangeal joints [54], and from femoral trochlear and condyles [44]) were sampled. Regarding bones, the groups of Boaks [53] and Pérez-Martínez [40] examined various long bones of fore and hind limbs, assuming equal degradation patterns across these bones. In fact, Boaks et al. [53] found a fore- versus hindlimb divergence in that the Co/NCo protein ratios were altered in humeral and in femural bone, reflecting the different amounts of cortical and cancellous bone in these skeletal elements. It is also indicated that varying changes in protein degradation rates occur in different muscles. Cell types (myocard, smooth- and skeletal muscle), pH and fiber type composition are reported as possible influencing factors [70,71,72,73]. Further quantitative studies, particularly those using human tissues, are required for validation.

#### 4.3.3. Applicability of Tissues in Forensic Case Work and Future Investigation

To achieve a method directly applicable at a crime scene entails a special requirement to target tissues. Although most tissues of the human body can be routinely collected during autopsy, sampling possibilities for field application are more limited, requiring low-invasive methods to be put into practice quickly and with less effort. In the selection of target tissues, priority should be thus given to availability and accessibility to enable controlled, sensible sampling. In this respect, tissues of the musculoskeletal system (bone, cartilage, and skeletal muscle) may be preferred over internal organs (e.g., heart, kidney, etc.). Musculoskeletal tissues are distributed all over the human body, and thus enable sampling even in dismembered body parts and in cases where injuries, onset of decomposition or insect inhabitation hinder access to specific sampling sites. Skeletal muscle in particular offers all these advantages, and is the most investigated tissue in protein-based PMI determination (8 low and moderate risk level 1 and 2 studies, Table S3).

### 4.4. Proteins

#### 4.4.1. Rate and Pattern of Protein Degradation

All analyzed studies clearly demonstrate that proteins degrade over time postmortem (Figure 6). Proteolytic enzymes and non-enzymatic processes (e.g., extreme pH values, temperature) interact to cleave proteins gradually into smaller fragments [14]. This fragmentation phenomenon has been consistently detected in forensic studies (Figure 6) and is confirmed by numerous studies of a non-forensic context (e.g., [19,71,74,75,76]). Moreover, the evidence from these studies indicates that decomposition rates vary between proteins investigated within the same study settings (i.e., when sampled from similar tissues). This suggests that individual proteins exhibit different susceptibility to postmortem proteolysis (Figure 6). The basis for this has been laid by studies in other scientific fields, including meat science (e.g., [76,77]) and neuropathology (e.g., [74,78,79]). Combined evidence indicates that the decomposition behavior of proteins is determined by a multitude of factors, such as differences in amino acid sequence, posttranslational modification (e.g., oxidation, nitrosylation, phosphorylation), all in turn influencing spatial structure, molecule function, and accounting for variability in protease cleavage sites [22,80,81].

#### 4.4.2. Degradation Kinetics of Individual Proteins

Troponin: The most frequently investigated proteins in PMI estimation belong to the troponin complex. Troponins are well known markers for myocardial injuries in clinical conditions [82] and for proteolysis in meat science [83,84]. In the forensic context, most groups investigated the degradation of the troponin subunit troponin T (TnT), but troponin I (TnI) was also tested for its suitability as a PMI marker. Regardless of study designs, cardiac TnT (cTnT) and cardiac TnI (cTnI) were found to degrade in similar patterns. Western blot experiments showed a time-dependent decrease and loss of the native cTnT and cTnI proteins, accompanied by the formation of distinct degradation products. Especially notable is a degradation product of cTnT with approximately 30 kDa, which was found in human heart and skeletal muscle [34,35,36,37,38,63]. The time of appearance of this degradation product is consistent with that reported for skeletal muscle degradation in cattle [85], pigs [63,70], lambs [76], chicken [86], and ducks [87], indicating a cleavage event conserved across many species, even if there has also been some incongruous evidence: In contrast to other work on porcine skeletal muscle, Foditsch et al. [55] found no degradation products of cTnT, but a continuing decrease of the native band. The reason for this is not finally clear, part of it may be found in the use of different antibodies. Since proteins often contain several epitopes to which commercial antibodies may bind, cleavage of epitope(s) can entail that the presence of smaller fragments is not recognized, resulting in different results depending upon the antibody product used.

The degradation of human cardiac TnI and TnT has been documented via Western blot analysis over PMIs up to 230 h [33,34,35,36,37]. In rats, the degradation of cTnI and cTnT has been followed via 2D-PAGE over 46 accumulated degree days (in short, an ADD of 46 °d), which corresponds to 48 hpm at 23 °C. During these periods, there was no loss of the native band, and no appearance of smaller fragments. However, immunolabelling data indicate that troponin degradation in cardiac muscle should be viewed with caution. Immunostaining of cTnT and cTnI in sections of canine, porcine, rat, and human heart muscle was found absent or significantly decreased after myocardial ischemia infarction [88,89]. Analogously, a porcine heart failure model using SDS-PAGE based Western blots showed that two months post-infarct cTnT and cTnI immunoreaction intensities were reduced by up to 70% [90]. Employment of cardiac troponins in PMI determination will, therefore, require the definition of appropriate exclusion factors.

In comparison to troponins in cardiac tissue, data on troponin degradation patterns in human and animal skeletal muscle appear more reliable. In humans, native cTnT remained detectable over 36 °d [38], and smaller fragments appeared at <28 and at 28 °d. In the pig, native cTnT exhibits a marked decrease at 110°d [55], and disappears significantly at a mean of ~201.6 °d [21]. Smaller degradation fragments of similar molecular weights as shown in humans were found at 105 and 115.5 °d [21,38]. The degradation pattern of troponin has provided crucial evidence contributing to the clarification of a forensic case [39]. Nevertheless, many studies on troponin degradation had to be associated with a high risk of bias, mostly due to flaws in the study design, hindering a consistent assessment of outcome. Further high quality studies, also investigating possible influencing factors, are required to validate and improve the knowledge of this promising candidate PMI marker.

Collagen: Due to its unique triple helical structure with strong inter- and intramolecular bonds, collagen displays low susceptibility to postmortem proteolysis [91]. This has been confirmed by several investigations and opens a perspective to be used as a late PMI marker. In human osseous remains in particular, collagens proved to be exceptionally stable. Several collagen types of human bones, including collagen I and collagen 5, remained detectable by mass spectrometry until up to 20 years [23] and Co/NCo ratio decrease in human bones proved trackable until 171 years postmortem [43]. Among 14 tested human collagen isoforms, only COL10A1 was completely lost in bones with a PMI longer than 12 years [23]. The collagen/non-collagenous (Co/NCo) ratio of buried porcine bones was found to decrease significantly (compared to fresh state) over a PMI of 12 month [53,56], and collagen type 5 decreased slightly over 6 months [65]. Overall, despite different designs, investigated species, methods and outcome analyzes, studies to date clearly highlight the qualification of collagens as late-PMI marker proteins.

Desmin: Postmortem proteolysis of the muscle-specific intermediate filament protein desmin has been documented for skeletal muscle of humans [38,45], pigs [21,55] and rats [45,49], and for cardiac tissue of rats [50]. Western blot analyzes have shown that with advancing PMI desmin is broken down into several smaller degradation products (mostly three fragments of <50 kDa). The present forensic studies confirm desmin degradation patterns already shown in meat science studies since the 1980s [84]. Degradation products similar to those formed in humans were confirmed to occur in skeletal muscle of cattle [92], lambs [76], and again pigs [63,93], just as in avian skeletal and smooth muscle tissue (chicken [71,94], duck [87,95], ostrich [96], goose [95]). A direct comparison of desmin degradation in skeletal muscle of humans, pigs, and mice was undertaken, again confirming the similarity of degradation events [63]. Similar to other relevant proteins, postmortal desmin degradation was found to follow species-specific time courses. Thus, the complete loss of the native protein occurred between 40 and 80 °d in rat skeletal muscle [45,49], but at ~170 °d in pig skeletal muscle [21]. For humans, no loss was observed in the studies included. The reason for this may mainly be attributed to the relatively short ADD (36 °d) of investigated human cases so far. Nevertheless, native desmin band loss in humans is strongly indicated by a study of Ehrenfellner et al., showing the absence of this band in a human muscle sample with a PMI of approximately 40 dpm [63]. The first degradation products of skeletal muscle desmin, with molecular weights between 41 and >35 kDa, appeared at a mean of 28.1 °d in humans [38], at 20 °d in rats [45,49] and, depending on study conditions, at 22 °d and ~40 °d in pigs [21,55], whereas the first degradation product of rat desmin had already disappeared again at 60 °d [49]. Further degradation products appeared between 19.3 and 110 °d. Those smaller fragments remained present until the latest investigated time points (up to 80 °d in rats [45,49], and 210 °d in pigs [21]). Desmin degradation products were found to appear consecutively (i.e., in no sample, the second fragment was present in the absence of the first one). The degradation patterns of desmin have already provided crucial evidence in the clarification a forensic case [39]. The consistency of the desmin findings is graded good, suggesting this protein can be used as a relevant marker of early and intermediate PMI estimation.

Tropomyosin: Postmortal disintegration of tropomyosin was found to depend on the source tissue. Tropomyosin from skeletal muscle was found to be stable over 36 °d in humans [38], and PMIs of 110 °d [55] and 210 °d [21] in pigs, and 80 °d in rats [49]. By contrast, tropomyosin from rat liver was found to decrease rapidly over a PMI of 48 hpm. Actual stability of skeletal muscle tropomyosin may extend beyond the reported PMI/ADD values, as the protein seems unaffected by proteolysis over the as yet investigated time spans (see above). Due to this stability, tropomyosin served as a control protein in a forensic case study [39]. Further investigations over longer PMIs are necessary. Also, it needs to be clarified whether the fast degradation of tropomyosin from rat liver also occurs in human liver samples and whether it may be induced by higher temperatures in the body core, or possibly influenced by the vicinity to the gastrointestinal tract.

Calpain: Two different methodological approaches were used to assess the degradation of calpains: Western blots to measure the relative concentrations of the native protein, and casein zymography to demonstrate the postmortem activity of these Ca^2+^-dependent enzymes. Due to the methodological differences, time-related data cannot be compared between studies. The subunits of calpain 1 (formerly µ-calpain) and calpain 2 (formerly m-calpain) are autolyzed in a consecutive order, resulting in the appearance of smaller forms [97]. This degradation pattern was observed in forensic studies on humans and pigs [21,38,39,55], and in a variety of studies in other fields [96,97,98,99]. In human muscle [38], calpain 1 activity was detected up to 36 °d (the longest PMI investigated). In porcine muscle, the activated (native) form of calpain 1 was detected up to ~76 °d [21]. With immunostaining, the native protein was in pigs present up to 110 °d [55]. An autolyzed form of calpain 1, appearing between ~17 and 39.6 °d, was found in humans and pigs [21,38,55]. Similar to desmin, the enzyme activity of calpain 1 and 2 already provided crucial evidence in the clarification a forensic case [39].

SERCA: Two pig studies provided similar results about postmortem cleavage of skeletal muscle derived sarco/endoplasmatic reticulum Ca^2+^- ATPase 1 (SERCA 1), showing degradation products between 100 and 35 kDa. A first degradation product appeared between 73.5 °d [55] and 147 d ° [21], a second degradation product at 165 °d [55] and ~201 °d [21]. The native protein remained detectable over the entire investigated PMI of 110 °d [55], but was lost at an average of ~210 °d in a longer-lasting study [21]. The SERCA 2 isoform was found to decline over PMIs of 105 °d and 441 °d in pigs [55] depending on storage temperature. More high-quality studies are required to expand the knowledge on SERCA degradation and to validate the results in human samples.

Titin and nebulin: Both proteins are exceptional in size, with molecular masses of 800–900 kDa (nebulin) and approximately 3.800 kDa (titin), and have thus mostly been analyzed only by SDS-PAGE (e.g., [55]). As titin and nebulin breakdown is associated with meat tenderness, multiple studies have investigated the postmortem degradation of these proteins in skeletal muscle of various livestock animals (e.g., [49,58,59]). In a forensic context, the postmortem behavior of titin and nebulin has so far only been investigated in animals. Native porcine titin was reported to degrade between ~12 °d [21] and 22 °d [55] into three smaller fragments. These appear between ~14 [21] and 22 °d [55], between ~61 [21] and 77 °d [55], and at ~177 °d [21], respectively. The native form of nebulin was found to be lost at 22 °d [55] and ~42 °d [21]. A second isoform of nebulin (N2) is described in the study of Pittner and co-workers [21] that disappeared at ~73.5 °d. Validation of protein identities by antibody labelling and testing of human isoforms is recommended.

Vinculin: Among included studies, postmortem degradation pattern of vinculin is only tested in animals yet. In rat skeletal muscle tissue, vinculin was found to degrade within a PMI of 80 °d [45,49]. The splice variant meta-vinculin [76] was completely lost at 60 °d. Vinculin is reported to cleave into two smaller fragments of 84 kDa and 75 kDa which appeared at 20°d and remained present over the investigated period of 80 °d [45,49]. In a small sample, vinculin degradation was also investigated in humans. Degradation was found to be similar as in the rat being characterized by the loss of meta-vinculin and the native vinculin band, and by the appearance of 4 degradation products [45]. The presently available forensic studies, thereby, confirm the degradation patterns of this protein shown in studies conducted in other contexts in various animals (chicken [94], mouse [100], sheep [76], pig [101]). Similar degradation behavior of vinculin also in human skeletal muscle may be expected, as indicated in a study of Ehrenfellner et al. [63] investigating muscle samples of three human cases with different PMI. Current knowledge suggests that vinculin is a promising candidate for protein-based PMI determination that should be further tested in an appropriate sample size in humans.

Alpha-actinin: In porcine skeletal muscle, alpha-actinin remained stable over PMIs of 110 °d [55] and 210 °d [21]. It remains to be tested whether this low susceptibility to proteolysis is also true for human muscle.

Laminin: Laminin 2 was found to be stable in human bones interred for up to 20 years [23] and the preservation of laminin without apparent decrease in porcine skeletal muscle over a PMI of 105 °d [55], proposes this protein as a late PMI marker. Further investigations are necessary to confirm its low proteolytic susceptibility, especially in soft tissues.

Creatine kinase: Postmortem degradation patterns of creatine kinase has as yet only been tested in animals. The degradation of the muscle type isoform was investigated using 2D-PAGE in rat hearts [50], and by mass spectrometry in porcine bones [57]. In heart, the enzyme was found to increase in amount during the early PMI (23 °d), followed by a decrease until 46 °d. In bone, the abundance of creatine kinase decreased strongly between 2 and 4 months postmortem, but the enzyme remained detectable until the latest sampling point at 6 months. As this protein was investigated in only two studies associated with a high risk of bias, more high quality research is necessary to validate and expand the findings to date and to test its suitability as a PMI marker.

β-catenin: The structural protein β-catenin remained stable up to 12 years in human bones [23] and demonstrated no postmortem changes over a PMI of 96 hpm in rat skeletal muscle [48]. This suggests the protein as a potential marker for the advanced PMI. Further research is necessary to confirm this.

Glutathione-S-transferase (GST): Postmortem degradation patterns of GST have as yet only been tested in animals. Information on the postmortem degradation of GST is heterogeneous. In the mouse liver, its activity was found decreased partially at 5.75 °d [58]. However, activity levels were found unaffected at later time points [58]. In rat liver, the 2D-PAGE spot intensities of GST mu 2 and GST alpha-5 increased over 46 °d, and decreased over 23 °d, respectively [50]. In the mouse kidney, GST activity increased over 46 °d, but no similar alterations were found for GST from skeletal muscle and brain [58]. Additional research to resolve these inconsistencies and further testing of human samples is required.

Calmodulin binding proteins: The degradation of the ubiquitous calmodulin binding proteins calcineurin (Cn) A, Ca^2+^/calmodulin-dependent protein kinase (CaMK) II and myristoylated alanine-rich C-kinase substrate (MARCKS) were investigated in skeletal muscle and lung tissue of rats [46] and mice [22] stored for 84 °d. No information of postmortem alteration of these proteins in human tissue is available so far. CnA was consistently found to decompose via a degradation product (present from 21 °d) and a marked decrease of the native protein band. MARCKs degraded with increasing PMI until it was almost lost at 84 °d. CaMKII was found to be stable over the investigated PMI in rat muscle (84 °d), whereas it was undetectable at 42 °d in mice. Further studies including human tissues are necessary to substantiate the findings in mice and rats, and to evaluate the suitability of these proteins as forensic PMI markers.

Peptide hormone: In human tissue, the postmortem stainability of the peptide hormones insulin, glucagon, calcitonin and thyroglobulin with specific antisera was found to be time-dependent. The temporal patterns of immunoreactivity were found to be roughly similar for insulin and glucagon. For insulin, one representative study [29] reports samples as stained (+) until 29 dpm, but some samples already as unstained (−) from 13 dpm onwards; a second study [41] identifies samples as (+) until 18 dpm, and as (−) at 22 dpm. Corresponding data for glucagon are (+) until 13 dpm, and (−) at 7 dpm [28], and (+) until 12 dpm, (−) at 8 dpm [41]). However, thyroglobulin and calcitonin were found to be highly susceptible to autolysis, resulting in the loss of immunostainability results already after only 1 dpm [41] or after approximately 5 dpm [31]. As noted in the study of Ortmann et al. [41], such variation may be partly explained by cohort differences in the autopsy cases used. Due to incomplete definition, differences in antibody specificity can also not be excluded. Within the early PMI range, positive control by simultaneous staining of fresh tissue could additionally help detect false negative staining (especially when loss of staining is present already at one day postmortem). As all studies investigating peptide hormones had to be associated with a high risk of bias, further high-quality research is required to substantiate the knowledge base on the postmortem degradation of these proteins.

#### 4.4.3. Postmortem Increase of Proteins

The majority of proteins were found to decrease postmortem. However, as already depicted above, some authors report increased protein levels after death, often followed by a subsequent decrease. The cause of the counterintuitive postmortem increase is often defined as unknown, although there are several possible explanations for this phenomenon. In very early postmortem phases, it could result from continued protein synthesis [22]. In this regard, Sanoudou et al. [102] reported a high transcriptional and possibly also translational activity during the first hours postmortem in skeletal muscle. Alternatively, a transient increase of a marker protein can in fact be the result of a degradation process, in that degradation products are stained together with the native protein. Moreover, postmortem accessibility of protein epitopes to antisera can increase [22], being directly manifested in increased immunostaining intensities. In distinct cases, the transient increase of a marker can also be evoked by the loss of live inhibition. Inhibitory units of enzymes can dissociate when membrane potentials break down, as exemplified by calpain activation due to increased postmortem Ca2+ levels. Finally, other methodological artifacts may also play a role. Thus, methods investigating the relative abundance of marker proteins compared to the overall protein/peptide concentration of a sample (e.g., Western blotting) may be biased in favor of the marker by high breakdown rates of other proteins.

#### 4.4.4. Applicability of Proteins in Forensic Case Work and Future Investigations

According to the current state of research, a protein-based future method should utilize a variety of proteins (deriving from one tissue), with different susceptibility to postmortem proteolysis (e.g., a set of predefined proteins, the transient presence of specific degradation fragments etc.) that can be compared to a pre-established database. Knowledge and consideration of timeframes in which degradation changes occur will facilitate the selection of such appropriate marker proteins (Figure 6). Proteins for an individual investigation may be selected depending on the PMI range to be examined.

## 5. Limitations of the Present Review

No meta-analysis could be conducted because of the high degree of inhomogeneity among the included studies regarding study design (time points, species, etc.) and outcome data. Although extensive effort was made to identify all relevant studies, it is possible that some have been missed because of non-availability in the searched databases, or incompatible terminologies. In addition, only research articles in English were considered. These may not represent all of the evidence.

## 6. Conclusions

Forensic research in recent years has presented numerous approaches aiming to supplement and/or substitute existing methods of time since death estimation. This adds to a complicated task because truly appropriate methods have to be capable of more than simply detecting a correlation between a measured postmortem marker and the PMI. Besides the fact that precision and reliability are essential aspects in time since death estimation [1], a new PMI estimation method, especially when considered for forensic routine case work, should as well be easy and fast in handling, and cost-effective in application. Combining these properties into one single method is difficult, which is probably the main reason why new biochemical approaches only rarely exceed basic research phases [103].

The present evaluation of the current research status on protein degradation-based forensic PMI determination provides a sound evidence base for the usefulness and sustainability of the general approach. Western blotting, mass spectrometry, immunohistochemistry, enzyme activity assay and casein zymography can be recommended as practicable methods of analysis, kidney, lung, bone, liver, and particularly skeletal muscle as appropriate sources of proteins with suitable degradation behavior. After all, the evidence base of at least 13 out of more than 130 considered proteins may be regarded as sufficiently proven. However, in view of the overall high numbers of tested methods (12), tissues (11) and proteins (more than 130), it can be considered unsatisfactory that an appropriate consistency of findings could only be found for two of the methods (Western blot, mass spectrometry), four tissues (lung, kidney, skeletal muscle, bone) and three proteins (collagen, desmin, tropomyosin). This is firstly due to the fact that studies were often associated with a high risk of bias, pleading for targeted study designs and precision of reporting. High-quality studies on human tissue in particular were rare and only exist for skeletal muscle (and in particular the proteins calpain 1 and 2, cardiac troponin T, desmin, and tropomyosin) and liver, investigated with Western blot, mass spectrometry and casein-zymography so far. Secondly, the still limited applicability can be explained by the fact that a large part of the studies have a “pilot character”, presenting novel approaches identifying new markers in new tissues with new methods. This together with the confirmation of the approaches’ principal validity and scope in applied field, can act as a clear encouragement to undertake advanced trials in this field, notwithstanding the constraints (e.g., costs/need for funding, time requirement, intermittently perhaps also dwindling ‘novelty’ and editorial interest in fast-moving times). Human studies, testing temporal sequences of postmortem degradation patterns of already available protein markers are urgently needed. With this, there is a good chance that the benefit will eventually outweigh the effort and provide forensic routine work with a powerful new diagnostic resource.

## Figures and Tables

**Table 1:** Study design of the 36 included articles. A animal, H human, pm postmortem, PMI postmortem interval.

Author and Year	Study Type	Tissue	Method	Model	Sample Size and Study Groups
Wehner et al., 1999 [29]	research article	pancreas	immunohisto-chemistry	H	*n* = 128 individuals with varying PMI
Wehner et al., 2000 [31]	research article	thyroid gland	immunohisto-chemistry	H	*n* = 147 individuals with varying PMI
Wehner et al., 2001 [30]	research article	thyroid gland	immunohisto-chemistry	H	*n* = 214 individuals with varying PMI
Wehner et al., 2001 [28]	research article	pancreas	immunohisto-chemistry	H	*n* = 136 individuals with varying PMI
Thaik-Oo et al., 2002 [32]	research article	brain, heart, liver, lung, kidney	enzyme-linked immunosorbent assay (ELISA)	H	*n* = 19 individuals with varying PMI
Kang et al., 2003 [46]	research article	lung, skeletal muscle	Western blot	A	*n* = 16 individuals; several groups/samples at different time points pm
Sabucedo and Furton, 2003 [33]	research article	heart	Western blot	A, H	animal: *n* = 3 hearts of 3 individuals; samples at different time points pm; human: 7 hearts of 7 individuals; several samples at different time points pm, samples of cases with varying PMI
Wehner et al., 2006 [27]	research article	brain, pancreas	immunohisto-chemistry	H	*n* = 500 individuals with varying PMI
Poloz and O’Day, 2009 [22]	research article	lung, skeletal muscle	Western blot	A	*n* = 40 individuals; several groups/samples at different time points pm, different temperature regimes
Boaks et al., 2014 [53]	research article	bone	histology/photometry	A	*n* = 12 bones of unknown (5?) individuals; several groups/samples at different time points pm
Bolton et al., 2015 [54]	research article	cartilage	Western blot	A	*n* = 33 trotters (individuals unknown); several groups/samples at different time points pm
Kumar et al., 2015 [35]	research article	heart	Western blot	H	*n* = 9 hearts of 9 individuals with varying PMI, several samples at different time points pm
Kumar et al., 2015 [34]	research article	heart	Western blot	H	*n* = 5 hearts of 5 individuals with varying PMI; several samples at different time points pm
Kumar et al., 2015 [36]	research article	heart	Western blot	H	*n* = 10 hearts of 10 individuals with varying PMI; several samples at different time points pm
Abo El-Noor et al., 2016 [47]	research article	heart, kidney	enzyme activity assay	A	*n* = 84 individuals; several samples at different time points pm
Lee et al., 2016 [48]	research article	kidney, skeletal muscle	immunohisto-chemistry, lateral flow assay, Western blot	A	kidney: *n* = 48 individuals, skeletal muscle: *n* = 40 individuals; several groups/samples at different time points pm
Li et al., 2016 [25]	review article		biuret method, immunohisto-chemistry, Western blot	A	n.i.
Foditsch et al., 2016 [55]	research article	skeletal muscle	sodium dodecyl sulfate polyacrylamide gel electrophoresis (SDS-PAGE), Western blot	A	*n* = 2 individuals; samples at different time points pm, different temperature regimes
Kumar et al., 2016 [37]	research article	heart	Western blot	H	*n* = 6 hearts of 6 individuals; samples at differnet time points pm of cases with varying PMI, different temperature regimes
Pittner et al., 2016 [21]	research article	skeletal muscle	casein zymography, SDS-PAGE, Western blot	A	*n* = 6 hind limbs of 3 individuals; several groups/samples at different time points pm
Pittner et al., 2016 [38]	research article	skeletal muscle	casein zymography, Western blot	H	*n* = 40 individuals with varying PMI
Kwak et al., 2017 [50]	research article	liver	2-dimensional (2D)-PAGE, mass spectrometry	A	*n* = 3 individuals; samples at different time points pm
Li et al., 2017 [42]	research article	liver	mass spectrometry	A, H	animal: *n* = 36 individuals, several groups/samples at different time points pm; human: *n* = 24 + 4 individuals
Li et al., 2017 [51]	research article	skeletal muscle	mass spectrometry	A	*n* = 4 individuals; samples at different time points pm
Ortmann et al., 2017 [41]	research article	pancreas, thyroid gland	immunohisto-chemistry	H	*n* = 105 individuals with varying PMI
Pérez-Martínez et al., 2017 [40]	research article	bone	mass spectrometry	H	*n* = 80 bones of 80 individuals with varying PMI
Pittner et al., 2017 [39]	case study	skeletal muscle	Western blot	H	*n* = 2 individuals with unknown PMI, forensic case
Jellinghaus et al., 2018 [56]	research article	bone	histology/photometry, histology	H	*n* = 16 bones (individuals unclear); samples at differnent time points pm
Prieto-Bonete et al., 2018 [23]	research article	bone	mass spectrometry	H	*n* = 40 bones of 40 individuals with varying PMI
Procopio et al., 2018 [57]	research article	bone	mass spectrometry	A	*n* = 8 bones of 4 individuals; samples at different time points pm
Zissler et al., 2018 [49]	research article	skeletal muscle	Western blot	A	*n* = 20 individuals; several groups/samples at different time points pm
Alibegovic et al., 2019 [44]	research article	cartilage	histology	H	*n* = 3
Cho and Eom, 2019 [52]	research article	lung	immunohisto-chemistry, Western blot	A	*n* = 45 individuals; several groups/samples at different time points
Choi et al., 2019 [45]	research article	skeletal muscle	mass spectrometry, Western blot	A, H	*n* = 20 (rat), *n* = 10 (mouse), *n* = 3 human
da Fonseca et al., 2019 [58]	research article	brain, liver, skeletal muscle, kidney	enzyme activity assay	A	*n* = 24–28 (unclear); several groups/samples
Jellinghaus et al., 2019 [43]	research article	bone	histology/photometry, histology	H	*n* = 35 forensic; *n* = 11 museum

**Table 2:** Overview of study details and outcome of the 36 included studies. pm: postmortem, dpm: days postmortem, hpm: hours postmortem, ADD accumulated degree days.

Author and Year	Method	Target (Species, Specific Tissue Location)	PMI	Samples and Sampling Frequency	Storage Conditions	Investigated Proteins	Type of Study Outcome	Main Study Outcome
Wehner et al., 1999 [29]	immunohistochemistry	human pancreatic α-cells	1–45 ± 1 dpm	128 samples; 1–13 samples per time point (41)	varying	Insulin	positive and negative immunostaining	loss of stainability over PMI
Wehner et al., 2000 [31]	immunohistochemistry	human thyroid gland	1–21 ± 1 dpm	147 samples; 1–20 samples per time point (21)	varying	Thyroglobulin	positive and negative immunostaining	loss of stainability over PMI
Wehner et al., 2001 [30]	immunohistochemistry	human thyroid gland	1–21 ± 1 dpm	214 samples; 1–20 samples per time point (21)	varying	Calcitonin	positive and negative immunostaining	loss of stainability over PMI
Wehner et al., 2001 [28]	immunohistochemistry	human pancreatic β- cells	1–21 ± 1 dpm	1–29 samples per time point (21); 1 sample per individual (136)	varying	Glucagon	positive and negative immunostaining	loss of stainability over PMI
Thaik-Oo et al., 2002 [32]	ELISA	human frontal lobe of the cerebrum, middle lobe of the right lung, apex of the heart, lower part of the liver, upper part of the kidney	2.75–120 hpm	19 samples per tissue; PMI between 2.75 and 120 hpm	varying	vascular endothelial growth factor VEGF	concentration of protein ng/mL	increase and subsequent decrease over PMI
Kang et al., 2003 [46]	Western blot	rat lung and skeletal muscle	0–96 hpm	4 samples per time point (4) per tissue	laboratory-controlled, 21 °C	calcineurin A (CnA), myristoylated alanine-rich C-kinase substrate (MARCKS), Ca^2+^/calmodulin-dependent protein kinase II (CaMKII), inducible nitric oxide synthase (iNOS)	band intensity, % of intact protein	significant decrease of band intensity over PMI; degradation products
Sabucedo and Furton, 2003 [33]	Western blot	bovine heart, human heart	bovine:0–6 dpm, “zero-hour” donor: 0–168 hpm; “PMI” donors: 8–12 hpm + 24 h incubation	animal: several samples at several time points, human: several samples of one individual at several postmortem time points + 12 samples of 6 individuals at different PMI	varying before autopsy; laboratory-controlled after autopsy, 20 ± 2 °C,	Troponin I (TnI)	band intensity, % of intact protein	pseudo-first order relationship between % of intact protein and log of time, decrease and fragmentation of protein over PMI
Wehner et al., 2006 [27]	immunohistochemistry	human frontal cortex and pancreas	1–23 ± 1 dpm	number of samples per time point is unknown; 1 sample per tissue per individual	varying	glial fibrillary acidic protein, somatostatin	positive and negative immunostaining	loss of stainability over PMI, temperature dependence (winter vs. summer)
Poloz and O’Day, 2009 [22]	Western blot	mouse lung and skeletal muscle	0–96 hpm	4 samples per time point (4) and per temperature (3)	laboratory-controlled; 5 °C, 10 °C, 21 °C	CnA, MARCKS, CaMKII, protein phosphatase 2A (PP2A)	band intensity, % of intact protein	significant decrease of band intensity over PMI; degradation products; significant effects of temperature
Boaks et al., 2014 [53]	histology/photometry	porcine long bones (extremities)	0–12 month pm	2 samples per time point (6)	outdoor; cadavers stored in kennels at surface of field, environmental conditions (e.g., temperature) unknown	Collagen	ratio of collagenous to non collagenous protein (Co/NCo) concentration	decrease in (Co/NCo) ratio
Bolton et al., 2015 [54]	Western blot	cartilage of porcine metacarpotarsal and metacarpophalangeal joints	0–6 weeks pm	2 samples per time point (7)	outdoor; buried in different soil environments, soil: 9–18 °C, air: 8–28 °C	Aggrecan	qualitative assessment of band presence/absence over PMI	loss of protein over PMI
Kumar et al., 2015 [35]	Western blot	human heart	unclear: probably up to 88.4 hpm	several samples at several time points	varying before autopsy; laboratory controlled after autopsy, 20 ± 2 °C	cardiac troponin T (cTnT)	band intensity, % of intact protein; migration distance of bands	significant decrease of band intensity over PMI, degradation products
Kumar et al., 2015 [34]	Western blot	human heart	approx. 5–230 hpm	several samples at several time points	varying before autopsy; laboratory controlled after autopsy, 20 ± 2 °C	cTnT	band intensity, % of intact protein; migration distance of bands	significant decrease of band intensity over PMI, degradation products
Kumar et al., 2015 [36]	Western blot	human heart	unclear: probably up to 88.4 hpm	several samples at several time points	varying before autopsy; room temperature after autopsy	cTnT	band intensity, % of intact protein; migration distance of bands	significant decrease of band intensity over PMI, degradation products
Abo El-Noor et al., 2016 [47]	enzyme activity assay	rat heart and kidney	0–7 hpm	12 samples per time point (7)	laboratory-controlled; 22 °C, 15% relative humidity	catalase, gluthatione-S-transferase, glutathione reductase	change of enzyme activity	significant decrease of enzyme activity over PMI
Lee et al., 2016 [48]	immunohistochemistry, lateral flow assay (LFA), Western blot	rat kidney and psoas muscle	0–96 hpm	5–6 samples per time points (8)	laboratory controlled; 23 ± 1 °C	glyceraldehyde-3-phosphat-dehydrogenase (GAPDH), caspase-3, peroxisome proliferator-activated receptor-γ (PPAR-γ), glycogen synthase, glycogen synthase kinase-3β (GSK-3β), p53, 5’ AMP-activated kinase α (AMPKα), beta catenin	WB: band intensity, % of intact protein, calculation of half maximum intensity of intact protein PMI_50;_ IHC: qualitative assessment of staining intensity; pilot experiments to develop LFA-based chip	WB: significant decrease of band intensity over PMI; IHC: loss of stainability over PMI; LFA-based chip: development of rGAPDH immunosensor
Li et al., 2016 [25]	Biuret method, immunohistochemistry, Western blot	rat cardiac muscle, brain, liver, lung, kidney, skeletal muscle, and spleen; human cardiac and skeletal muscle; rabbit skeletal muscle	48 hpm, 54 hpm, 72 hpm, 5 dpm, 7 dpm	not indicated	not indicated	actin, tubulin, myoglobin, troponin I	not indicated, varying	not indicated, varying
Foditsch et al., 2016 [55]	SDS PAGE, Western blot	porcine biceps femoris muscle	4 °C: 0–21 dpm, 22 °C: 0–5 dpm	1 sample per temperature and per time point	4 ± 1 °C, 22 ± 2 °C	α-actinin, calsequestrin 1, desmin, nebulin, titin, sarcoplasmic/endoplasmic reticulum Ca2+ ATPase-1 (SERCA-1), SERCA-2, tropomyosin, cTNT, laminin, µ-calpain	qualitative assessment of band presence/absence over PMI	loss of proteins over PMI, degradation products
Kumar et al., 2016 [37]	Western blot	human heart	unclear: probably up to 189 hpm	6 individuals; several samples at several time points and temperatures	12 °C, 20 ± 2°C, 25 °C, 37 °C	cTnT	not defined, probably % of intact protein	significant decrease/loss of intact protein over PMI, degradation products, effect of temperature
Pittner et al., 2016 [21]	casein zymography, SDS PAGE, Western blot	porcine biceps femoris muscle	0–10 dpm	6 samples per time point (17)	21 ± 1 °C, 35% relative humidity	titin, tropomyosin, nebulin, desmin, cTNT, SERCA-1, capain-1, calpain-2	mean time points (+ 95% confidence interval) of band change (presence to absence and vice versa)	different mean time points of band change of different proteins and degradation products
Pittner et al., 2016 [38]	casein zymography, Western blot	human vastus lateralis muscle	4–93 hpm	40 samples at differnet time points	varying, accumulated degree days calculated	desmin, tropomyosin, calpain-1, calpain-2	presence and absence probability of bands at different accumulated degree days; correlation of band presence and absence with ADD	different probability of presence for different proteins and degradation products over ADD; significant correlations between proteins/degradation products and ADD
Kwak et al., 2017 [50]	2D-PAGE	rat liver and heart	0–48 hpm	3 samples per time point (3)	laboratory-controlled; 23 ± 3 °C, relative humidity 60 ± 5%	listed in supplementary material	spot intensity	increase and decrease of spot intensity over PMI
Li et al., 2017 [42]	mass spectrometry	rat liver, human right posterior liver lobe	rats: 0–144 hpm, human: estimated PMI + 0–144 hpm (corresponds to 10–168 hpm)	several samples per time point (4)	laboratory-controlled; 23 ± 1 °C, 30–45% relative humidity	listed in supplementary material	signal strength of peptide/protein peaks	decrease of signal strength of various protein/peptide peaks over PMI
Li et al., 2017 [51]	mass spectrometry	rat quadriceps femoris muscle	0–144 hpm	1 sample per time point (4)	laboratory controlled; 23 ± 1 °C, 30–45% relative humidity	listed in supplementary material	signal strength of peptide/protein peaks	decrease of signal strength of various protein/peptide peaks over PMI
Ortmann et al., 2017 [41]	immunohistochemistry	human pancreas, human thyroid gland	up to 22 dpm	1–18 samples per time point (12)	varying	calcitonin, thyroglobulin, insulin, glucagon	positive and negative immunostaining	loss of stainability over PMI
Pérez-Martínez et al., 2017 [40]	mass spectrometry	human femur, tibia and humerus	5–47 years pm	80 samples at different time points	Outdoor; cemetery, winters 5–19 °C, summers 22–40 °C	collagen type I	abundance of proteins (number of peptides)	significant decrease of collagen concentration over PMI
Pittner et al., 2017 [39]	Western blot	human vastus lateralis muscle	unknown	1 sample per individual	partly unknown	desmin, troponin T, calpain 1, calpain 2, tropomyosin	presence and absence of bands in two individuals to trace progression of events in murder-suicide case	difference (presence and absence of proteins) in protein profile of two individuals
Jellinghaus et al., 2018 [56]	histology/photometry, histology	porcine right and left femur	0–3 month pm	8 samples per time point (4)	buried in boxes; 13–34 °C (monitored); 2 groups with different (hay and distilled) water infusion	collagen	ratio of collagenous to non collagenous protein (Co/NCo) concentration	decrease in (Co/NCo) ratio
Prieto-Bonete et al., 2018 [23]	mass spectrometry	human proximal femur	5–20 years pm	40 samples at differnet time points/PMI	outdoor; cemetery, weather data available online	listed in supplementary material	presence and absence of proteins at different PMI	loss of proteins over PMI
Procopio et al., 2018 [57]	mass spectrometry	porcine tibia	0–1 year pm	1 respectively 4 samples per time point (5)	outdoor, buried in soil, temperature data not available	listed in supplementary material	abundance of different proteins at different PMI	decrease in protein amount with PMI
Zissler et al., 2018 [49]	Western blot	rat quadriceps femoris muscle	0–4 dpm	4 samples per time point (5)	laboratory-controlled; 20 °C	desmin, vinculin, tropomyosin	change of band pattern (presence and absence of bands) over PMI; band intensity in % of intact protein	decrease of band intenstiy over PMI; significant loss of protein bands over PMI; degradation products
Alibegovic et al., 2019 [44]	histology	cartilage of human trochlea, medial and lateral condyle	estimated PMI (30–48 hpm) + 1–36 dpm	3 samples per time point (3) and per temperature	varying before autopsy; laboratory-controlled after autopsy, storage of samples in tubes, 11 ± 2 °C, 35 ± 2 °C	collagen, proteoglycan	intensity of histological staining; Bern grading scale	significant decrease in staining intensity over PMI; no significant difference between temperatures
Cho and Eom, 2019 [52]	immunohistochemistry, Western blot	rat lung	1–7 dpm	5 samples per time point (7)	laboratory-controlled; immersion in sea water, 15 ± 5 °C	RAGE	Western blot: band intensity in reference to housekeeping protein (GAPDH); immunohistochemistry: qualitative assessment of staining intensity	decrease of band intensity over PMI; decrease of IHC staining intensity
Choi et al., 2019 [45]	mass spectrometry, Western blot	mouse, rat, human vastus lateralis muscle	0–96 hpm	mouse: 2 samples per time point (5); rat: 2 samples per time point (5) for MS, 5 samples per time point (5) for WB, human: 3 samples at differnt time points	laboratory-controlled in rat: 20 °C, mouse: 25 °C; human: varying	eukaryotic translation elongation factor 1A2 (eEF1A2), GAPDH, tropomyosin, desmin, vinculin	MS: decrease of abundance over PMI, WB: change of band pattern (presence and absence of bands) over PMI	MS: decrease of proteins over PMI; WB: significant loss of bands over PMI; degradation products
da Fonseca et al., 2019 [58]	enzyme activity assay	mouse liver, whole brain, gastrocnemius muscle, kidney	0–48 hpm	6–7 samples per time point (4)	laboratory-controlled; 22 ± 2 °C	Na+/K+ ATPase, Acethylcholinesterase, gluthatione-S-transferase	change of enzyme activity	significant increase and decrease of enzyme activity over PMI/at time points pm
Jellinghaus et al., 2019 [43]	histology/photometry, histology	human femur	up to 171 years pm	46 samples at different time points	outdoor; cemetery and archeological samples (museum)	collagen	ratio of collagenous to non collagenous protein (Co/NCo) concentration	decrease in (Co/NCo) ratio

## Supplementary Materials

The following are available online at https://www.mdpi.com/2075-4418/10/12/1014/s1, File S1. Instructions and questions to assess the risk of bias, File S2. Template to obtain the overall risk of bias, File S3. Instructions to assess the strength of the body of evidence, File S4. Summary and critical analysis of the included studies in historic context, Table S1. List of proteins included in all included articles, Table S2. Results of the assessment of the risk of bias for all included articles, Table S3. Results of the assessment of the evidence base for different tissues, methods and proteins.
