# Supplementary material for: Postmortem Protein Degradation as a Tool to Estimate the PMI: A Systematic Review
Source: Diagnostics (Basel). 2020 Nov 26;10(12):1014. doi: 10.3390/diagnostics10121014 (PMC7760775; doi:10.3390/diagnostics10121014)
Supplement: Supplementary file 1 [file diagnostics-10-01014-s001.zip › diagnostics-989370-File S3.pdf]

diagnostics

Assessment of the strength of the body of evidence

To assess the strength of the body of evidence of the current research status, the authors used the 'NHMRC approach to grade evidence recommendations' [1] as a template to establish a grading matrix for the non-clinical studies included in the review. The following components were evaluated:

1. the **evidence base** in terms of the number of studies, level of evidence and quality of the study (risk of bias)
2. the **consistency** of study results
3. the **generalizability** of the body of evidence to the target population

1. Evidence base

To assess the evidence base of study outcomes, the number of studies, the level of evidence and the assigned risk of bias of individual studies were taken into account. The quantity of evidence was rated by the available number of included studies as the evidence base for a specific outcome. A high level of evidence was assigned to the most relevant study design contributing to the development of a PMI estimation method (Figure 1). A low level of evidence was assigned to a study design that is less robust to answer the study question and thus considerably contributes less to the methods' development. The quality of evidence was rated by taking into account the assigned risk of bias of individual studies.

The "level of evidence" is assigned according to study design and investigated species. Studies using a number of individuals allowing statistical analysis were allocated quantitative studies with a high statistical power. The sample size had to be a minimum of 4. Case studies and pilot studies with lower sample sizes (n<4) are less robust to answering study questions and are therefore assigned a lower "level of evidence". Additionally, investigating human tissue is rated with a higher "level of evidence" in respect to developing a PMI estimation method compared to animal studies:

Level I: quantitative human study (n≥4 individuals)
Level II: human case study (n<4 individuals) or quantitative animal study (n≥4 individuals)
Level III: animal (pilot) study (n<4 individuals)

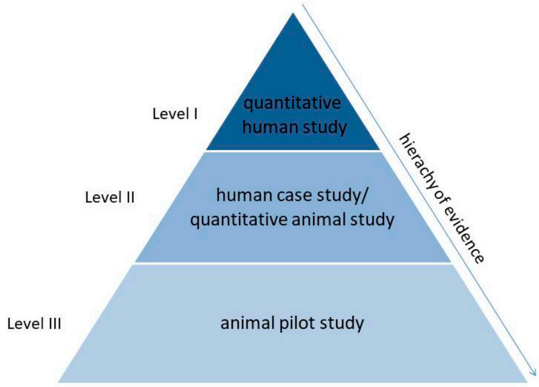

Figure 1. Different study designs were associated with different evidence hierarchies from level I to level III.

2. Consistency

To assess the consistency of evidence, the extent to which the findings were consistent across the included studies, across the range of study populations and study designs was evaluated. This allows evaluations of whether the results are replicable or only likely to occur under specific conditions. The

evidence of consistency of study results was applied for methods, tissues and proteins that were investigated in at least 3 studies with a low or moderate risk of bias.

3. Generalizability

To assess the generalizability of results, we evaluated how precisely the available evidence answered the respective research question (e.g. is protein degradation suitable to estimate the PMI). We considered how well the selected cases matched the population being targeted in practice. As the generalizability of evidence is only relevant for humans in routine practice, only studies investigating human tissues were included for assessment.

Rating and descriptions of the evidence base, consistency and generalizability assessment:.

Component	Excellent	Good	Satisfactory	Poor
**Evidence base**	>2 low risk of bias level I or level II studies	1-2 low risk of bias level I or II studies AND >1 moderate level I or II	1-2 low risk of bias level I or II studies OR >1 moderate level I or II	Else
**Consistency**	All studies consistent	Most studies consistent, inconsistency can be explained	Some inconsistencies	Evidence is inconsistent
**Generalizability**	Population/s studied in body of evidence are the same as the target population in the guideline	Population/s studied in the body of evidence are similar to the target population for the guideline	Population/s studied in the body of evidence differ to the target population guideline but it is clinically sensible to apply this evidence to the target population	Population/s studied in the body of evidence differ to the target population and hard to judge whether it is sensible to generalize to target population

References

1. National Health and Medical Research Council NHMRC levels of evidence and grades for recommendations for guideline developers. **2009**, Available from: https://www.nhmrc.gov.au/_files_nhmrc/file/guidelines/developers/nhmrc_levels_grades_evidence_120423.pdf.
