# Supplementary material for: Postmortem Protein Degradation as a Tool to Estimate the PMI: A Systematic Review
Source: Diagnostics (Basel). 2020 Nov 26;10(12):1014. doi: 10.3390/diagnostics10121014 (PMC7760775; doi:10.3390/diagnostics10121014)
Supplement: Supplementary file 1 [file diagnostics-10-01014-s001.zip › diagnostics-989370-File S4.pdf]

diagnostics

MDPI

Summary and critical analysis of the included studies in historic context

From 1999 to 2001, the group of Wehner et al. chose immunohistochemistry (IHC) of human pancreas and thyroid gland as a means for protein detection to improve PMI estimation, resulting in a total of 4 studies. Staining characteristics of the peptide hormones insulin [1] and glucagon [2], and thyroglobulin [3] and calcitonin [4], were found to vary depending on the PMI and external conditions. Pancreatic β-cells were invariably positive for insulin until a PMI of 12 dpm, but had lost all stainability for insulin at a PMI of 30 dpm or longer. Regarding glucagon, a positive reaction was present in all cases with a PMI of up to 6 dpm, while a complete loss was found from 14 dpm onwards. Thyroglobulin and calcitonin remained stainable up to a PMI of 5 dpm and 4 dpm, respectively, and remained both unstained from a PMI of 13 dpm onwards. Although the number of tested individuals was high (between 128 and 214), all studies were assigned with a high risk of bias because they lack detailed description of measurement procedures and data analysis.

Thaik-Oo et al. (2002) [5] introduced the enzyme-linked immunosorbent assay (ELISA) technique into the field of forensic PMI estimation. Investigating the degradation of vascular endothelial growth factor (VEGF) in various organs (brain, kidney, heart, lung, liver) of 19 individuals with PMIs ranging from 2.75 to 120 hpm, these authors found largely time-variant but partly organ-dependent responses. In brain, kidney, lung and liver, a linear increase of VEGF concentration levels at early PMIs was followed by a decrease at PMIs after 24 hpm. By contrast, no correlation between VEGF concentrations and PMI was found in cardiac muscle. The study was associated with high risk of bias, mainly due to missing outcome data and deficits in reporting.

In 2003, western blot analysis entered the spectrum of PMI estimation methods. Using gel-electrophoresis based western blotting, Sabucedo et al. [6] tested cardiac troponin I (cTnI) for its suitability as a PMI estimation marker. In one experiment, the authors found that intact bovine TnI protein fragmented with progressing postmortem time. For a second experiment, they analysed human heart muscle from 6 autopsy cases with varying PMI and unknown storage conditions. Specimens were analyzed at the time of sampling and after additional 24 h incubation at room temperature. As in the bovine heart, a time-dependent fragmentation of the cTnI protein was detected. In an attempt to quantify the degradation, percentages of intact cTnI protein were calculated for different time points, although without reporting calculation details and significance levels. Because of these deficits, and low number of samples, also this study was assigned with a high risk of bias.

Kang et al. (2003) [7] used western blots to investigate the degradation of calmodulin-binding proteins in lung and skeletal muscle of 16 rats. Sacrificed animals were stored at 21°C and tissues were sampled at 0, 24, 48 and 96 hpm. It was found that calcineurin A (CnA) significantly degraded in a predictable pattern (via a degradation product) in the lung, while it produced unpredictable band patterns in skeletal muscle. $Ca2+$/CaM-dependent kinase II (CaMKII) remained stable within the investigated PMI of 96 hpm in both lung and muscle. In the lung, also myristoylated alanine-rich C-kinase substrate (MARCKS) exhibited a steady pattern of decrease, whereas bands of inducible nitric oxide synthase (iNOS) were irregular and largely unpredictable. Although the study is generally well designed, details about the sampling procedure and site are lacking, leading to a moderate quality judgement.

Poloz and O'Day (2009) [8], also using SDS-PAGE based western blotting, investigated a largely similar set of proteins in lung and skeletal muscle of mice. Animals were stored at temperatures of 5 °C, 10 °C and 21 °C and sampled at 0, 24, 48 and 96 hpm. In lung tissue, CnA turned out to be almost fully degraded after 24 hpm even when stored at low temperature (5 °C), CaMKII was undetectable by 48 hpm at all temperatures, and MARCKS was found to be significantly degraded at 96 hpm at 10 °C and 21 °C. Phosphatase 2A (PP2A) remained unchanged until 96 hpm at 5 °C but exhibited significant decreases at 96 hpm at all higher temperatures, after interim increases at 24 and 48 hpm. In skeletal muscle, CnA underwent a rapid cleavage into smaller fragments, and the native band became significantly weaker within the first 24 hpm. CaMKII was undetectable by 48 hpm, and

MARCKS was not detectable in any of the samples. PP2A was found to be stable for up to 96 hpm at 5 °C and 10 °C, while a marked decline after 48 hpm was found at 21°C. A statistically significant effect of temperature was also confirmed for CnA, with faster degradation of both, the native protein and the degradation product at higher temperatures. Although the study is in general ~~was~~ well designed, details about the sampling procedure and the sampling site are lacking, which resulted in a judgement of moderate quality.

In 2006, Wehner et al. [9] added to and expanded their earlier immunohistochemical research, now including two different proteins from two human organs: glial fibrillary acidic protein (GFAP) from the brain, and somatostatin from pancreas. Stainability of GFAP in the tissue of the frontal cortex persisted until 3 dpm, and no staining was consistently found after 14 dpm. Pancreatic somatostatin was constantly detected between 1 and 2 dpm, but its stainability was consistently lost at PMIs of 11 dpm or longer. In addition, a seasonal temperature dependence of degradation was detected for both proteins, with decomposition being faster in the warmer months of the year and slower in winter. Although the study was based on a large number of human cases (n = 500), it had to be associated with an overall high risk of bias due to a lack of a detailed description of measurement procedures and data analysis.

Boaks et al. (2014) [10] used a porcine model to test whether the ratio of collagenous and non-collagenous (Co/NCo) protein concentrations in skeletal remains could serve as a [new] measure for PMI estimation. Therefore, pig carcasses were left to decompose in cages on the surface of a field, and long bones were recovered and analysed after 2, 4, 6, 10 and 12 month, using fresh bones as controls. Dried standardized cross sections of epoxy resin soaked limb bone diaphyses were stained with a combination of sirius red and fast green (visualizing Co-proteins pink and NCo-proteins green, respectively), and the stains subsequently eluted. Concentrations of Co and NCo protein were photometrically analyzed from the eluates. The result was an overall decrease of the ratio of Co/NCo concentration over the investigated PMI (negative correlation between the Co/nCo-ratio and time; r = 0.563). In addition, these authors reported that bones degraded non-uniformly, with first signs of collagen loss appearing in the endosteal and/or periosteal regions, whereas mesosteal regions retained their collagen for longer time. Due to the small sample size (2 bones per postmortem time point), also this study was assigned to be of high risk of bias.

Similar to Sabucedo and Furton [6], the group of Kumar et al. published in 2015 and 2016 a sequence of four studies [11–14] on the postmortem degradation of human cardiac troponin T (cTnT), another member of the troponin protein family. The analyzed heart tissue derived from forensic cases with different background: cases without a prior history of disease (n = 6) [11], cases of fatal burn (n = 9 [12], and n = 10 [14]), and cases of electrocution (n = 5) [13]. SDS-PAGE based western blot analysis revealed that native cTnT degraded in a pseudo-linear relationship between percent of intact cTnT and PMI, and that cTnT over time cleaved into several degradation products. The authors also demonstrated that cTnT decomposition is temperature-dependent, with increased degradation rates at higher temperatures. Data on incubation times and numbers of samples per storage temperature are not provided. All studies were rated with a high risk of bias because of small sample sizes, deficits in outcome reporting and probable multiple use of original data.

Bolton and co-workers (2015) [15] used 33 pig trotters to investigate cartilage degradation for its suitability for the purposes of time since death estimation. Similar to Boaks et al. [10], specimens were buried in pre-prepared graves and recovered at defined time points postmortem (weekly for 6 weeks). Fresh trotters were used as controls. Each week, cartilage of the metacarpophalangeal and metatarsophalangeal joints was sampled and analyzed by western blotting. As a main result, the cartilage specific proteoglycan core protein aggrecan was found to degrade within the investigated PMI, the native band disappearing from 30 dpm onwards. A statistical analysis was not presented. Because of this and due to the small number of samples the study was associated with high risk of bias.

Abo El-Noor et al. (2016) [16] used enzyme activity assays to investigate the postmortem activity of glutathione-S-transferase (GST), glutathione reductase (GR) and catalase (CAT). They compared samples of heart and kidney tissue of 84 rats taken at the time of death and every following hour up

to 7 hpm. Enzyme function decline turned out to be organ-dependent, being faster in the heart than in the kidney. Thus, activities of heart CAT and GT were already significantly reduced at 1 hpm, and that of heart GST at 2 hpm. In kidney, by contrast, it took until 3 hpm that significant activity declines were found for GR and GST, and even until 6 hpm for CAT. During the investigated PMI of 7 hours, none of the investigated enzymes exhibited a re-increase in activity. For GR, a significant correlation between the activity decrease and PMI was found in both tissues. The study was associated with low risk of bias.

The groups of Foditsch et al. (2016) [17] and Pittner et al. (2016) [18] used SDS-PAGE, SDS-PAGE based western blotting and casein zymography (only in Pittner et al.), to trace PMI-dependent protein degradation patterns in porcine hindlimb muscle. The study of Foditsch [17] and co-workers is of pilot character, using only one animal each in two thermal regimes. Whole pigs were stored at 4°C (± 1 °C) and at 22 °C (± 2 °C), and muscle samples (M. biceps femoris) were taken through periods of 21 days and 5 days, respectively. Pittner et al. [18] investigated protein decomposition in 6 amputated pig hindlimbs stored for 10 days at 21 ± 1 °C and a mean humidity of 35 ± 5%. Samples were dissected from biceps femoris muscle at 17 predefined time points. Both studies investigated a largely similar set of proteins including α-actinin, calsequestrin 1, desmin, nebulin, SERCA1, SERCA2, titin, tropomyosin, cTnT, laminin, and calpain. The two studies uniformly demonstrate that some of the tested proteins degrade in a regular and predictable time-dependent fashion. Degradation products present in different postmortem time frames were found for the proteins titin, desmin, SERCA1, nebulin, and cTnT. At room temperature, tropomyosin and α-actinin proved stable over 5 [17] respective 10 [18] days, calsequestrin 1 and laminin over 5 days [17]. These four proteins were also present unchanged in all samples stored for 21 days at 4°C [17]. Notably, the quantification strategy utilized by Pittner et al. diverges from that applied in several previous work. Instead of comparing percentages of band intensities [6–8,11], the authors focused on the timing of appearance and disappearance of the SDS gel bands of the native proteins and their degradation products, calculating mean time points (+95% confidence interval) of band changes (presence to absence and vice versa). This allows defining specific time intervals in which individual proteins and their degradation products are either significantly present or absent. Despite the reasonable study design, this study was rated to be of moderate risk of bias as a detailed description of the assessment of band visibility is missing. The work of Foditsch et al. was assigned with a high risk of bias, mainly because of the small sample size.

Follow-up work of the group of Pittner and coworkers, again using SDS-PAGE based Western Blotting and casein zymography, continued and expanded their investigation on the degradation profiles of cTnT, desmin and tropomyosin, this time testing *M.* vastus lateralis samples from 40 human forensic cases with known PMI [19]. Results are presented in the form of statistically calculated probabilities of presence and absence of protein bands with increasing accumulated degree days (ADD), employed as a combined measure of PMI and environmental temperature. This enabled to draw conclusions on the time frame in which a particular protein is present with high probability. Thus, while tropomyosin was present in all investigated cases regardless of ADD, cTnT and desmin presented distinct decomposition events (loss of native band, appearance of degradation products) associated with a presence/absence probability in different time frames. Factors with possible influence on protein degradation dynamics, such as age, sex, and body weight (as represented by BMI) were additionally tested. Results indicate relevant influence of age and BMI, while sex and cause of death appear to have no major effect. In 2017, the methodology developed in this study was for the first time successfully applied to investigate on a criminal case [20]. It was possible to proof that two individuals found dead in a lake died un-simultaneously, thus confirming a certain succession of events determined *by* the criminal investigators.

The scientific basis of the subject matter was further broadened by the review article of Li et al (2016) [21], summarizing the research progress on PMI estimation made by the Chinese forensic community between 2004 and 2008. Six studies originally published in Chinese were reported. Together, they investigated the degradation of four proteins (actin, TnI, myoglobin, and tubulin) in various tissues of animal models and humans using IHC, western blotting and the Biuret method, a

copper salt-based colorimetric technique specific for proteins and peptides. The latter method was employed to evaluate the myofibril fragmentation index of homogenized skeletal muscle samples. The majority of the studies demonstrated individually distinct degradation patterns over the investigated PMIs. Accordingly, the authors concluded that the degradation of specific proteins correlates significantly with the PMI, however with the reservation that application in forensic practice is complicated by the fact that the degradation process is affected by additional factors of influence.

At around the same time, Lee et al. [22] implemented a multi-methodological approach combining western blotting, IHC and a lateral flow assay (LFA) for time since death estimation. Samples of rat psoas muscle and kidney were taken at specific time points between 0 and 96 hpm and analyzed for the degradation of glyceraldehyde 3-phosphate dehydrogenase (GAPDH), caspase-3, peroxisome proliferator-activated receptor gamma (PPAR-γ), glycogen synthase (GS), Glycogen synthase kinase-3 β (GSK-3β), p53, AMP-activated protein kinase α (AMPKα) and β-catenin. For most of these proteins, high correlations of degradation with the PMI were found. Calculation of PMI_{50} (half maximum intensity of intact protein) values allowed to classify the investigated proteins into short-term markers (PMI_{50} within 24 hpm), mid-term (PMI_{50} between 24 and 96 hpm) and long-term markers (PMI_{50} later than 96 hpm). AMPKα, GSK-3β, GS and caspase 3 from psoas muscle were identified as 'short term', renal caspase 3, GAPDH and PPAR-γ as 'mid-term', and p53 and β-catenin as 'long term'. The degradation of GS and caspase-3 was additionally investigated by IHC. Renal GS was found to remain stainable within 24 hpm, but was no longer detected from 48 hpm onwards. Stainability of caspase-3 was completely lost at 96 hpm. In muscle, there was no staining for both proteins at 96 hpm. In an additional pilot approach, an LFA-based diagnostic chip was tested for its suitability to detect intact GAPDH in rat kidney in future forensic field application. The western blot approach within this study was associated with a low risk of bias. The IHC part was assigned with a moderate risk of bias as a detailed description of the assessment of staining intensities is missing, the LFA-approach with high risk.

Ortmann et al. (2017) [23] revisited the immunohistochemical approach by Wehner et al. [1–4,9], investigating the degradation of proteins from thyroid gland and pancreatic tissue of 105 human autopsy cases with different PMIs (ranging from several hours to 22 days), causes of death, and a broad spectrum of environmental conditions before autopsy. The authors found that the immunostainability for insulin, glucagon, thyroglobulin and calcitonin was time-dependent. For thyroglobulin and calcitonin, a weak stainability persisted for up to 8 days in some cases, but was already lost at 1 dpm in others. Insulin and glucagon could always be labelled to a PMI of 18 and 12 days, respectively, while all immunoreactivity for these proteins was lost from day 22 and 18 onwards. The study was assigned with a high risk of bias, mostly due to a lack in describing detailed measurement procedures and data analysis.

In 2017 and 2018, the groups of Kwak [24], Li [25,26], Pérez-Martínez [27], Procopio [28] and Prieto-Bonete [29] introduced proteomic approaches for time since death estimation.

Kwak et al. (2017) [24] specifically used HPLC-MS (high performance liquid chromatography mass spectrometry) to trace the degradation of 26 proteins from rat heart and liver separated by two dimensional polyacrylamide gel electrophoresis (2D-PAGE). Whole organs of 3 animals were dissected immediately after death and stored under controlled conditions in plastic containers. Tissues were sampled at 0, 24 and 48 hpm. Intensity measurements of protein spots in the polyacrylamide gels presented a heterogeneous picture, revealing either increase or decrease over the 48 hours PMI (individual proteins are listed in Table S1). Although the study is generally well designed, it had to be rated with a high risk of bias due to a low sample size (one animal per time point) and consequently, lack of statistical analysis for PMI correlation.

The two studies of Li et al. employed liver [25] and skeletal muscle [26] to assess the applicability of MALDI-TOF-MS (Matrix Assisted Laser Desorption/Ionization Time of Flight Mass Spectrometry) for PMI estimation. Rat cadavers (n = 36 for liver, n = 4 for skeletal muscle) and human liver lobes (n=24) were stored under controlled conditions, and samples dissected at different time points. Intensities of numerous peptide/protein peaks from both tissues were found to change significantly

over the investigated PMI. Especially, peaks with mass-to-charge ratios between 2826 and 1596 (rat liver), 3336 and 453 (human liver), and 1564 and 2168 (rat muscle) decreased markedly in intensity over the sampled time and were therefore suggested as appropriate markers for PMI estimation. On the basis of 24 in vitro decomposed human liver samples, various classification models were designed and validated using four additional cases. The two studies are in general well designed. Especially the study using liver tissue [25] is rated with a low risk of bias. Due to the small sample size (only one sample per time point), the second study using muscle tissue [26] was associated with high risk of bias.

Procopio et al. [28] used four pig carcasses to search for specific biomarkers that can [could] aid in PMI delimitation. The animals were buried, and after 1, 2, 4 and 6 months, one of the carcasses was partially excavated and one tibia was collected. The second tibia of each animal was collected at a PMI of 1 year. Using liquid chromatography mass spectrometry/mass spectrometry (LC-MS/MS), 28 proteins were initially found to be more abundant in samples collected after a PMI of one month than at the later times. After methodological refinements, the authors focused on the degradation of 18 proteins, including plasma proteins, muscle proteins, and proteins involved in glycolysis and gluconeogenesis pathways (all listed in Table S1). While some proteins decreased rapidly, others showed a lower depletion rate, until a rapid general decline in abundance occurred after 4 months. Only little further changes followed between 4 and 6 month. After one year, only 29% of the proteins could yet be analyzed, 10 of which showed inter-animal differences and 9 proteins not reaching significant differences. For the protein biglycan, the authors additionally report a statistically significant increase of deamidation with increasing PMI. Despite a satisfactory study design, the pilot character (1 animal per time point) led to a judgment of high risk of bias.

Pérez-Martínez et al. [27] and Prieto-Bonete [29] both investigated protein decay in human bones exhumed from a cemetery in the southeast of Spain. Pérez-Martínez et al.[27] used HPLC-MS/MS to assess the content of several proteins (and of non-protein biomolecules) in samples of 80 long bones of individuals interred for 5 to 47 years. The amount of collagen type I was found to be significantly lower in bones with a PMI of ≥ 20 years compared to bones with shorter PMI. Mathematical modeling indicated a reliability of roughly 70%. Prieto-Bonete [29] used LC-MS/MS to analyze femur bone samples of 40 humans interred for 5 to 20 years. Multiple correspondence analysis identified 32 proteins that allow to discriminate skeletal remains with a PMI of 5-12 years from those with a PMI of 13-20 years (proteins listed in Table S1). 16 of the proteins, including those with high molecular weights, were only present in samples with PMIs <12 years. The remaining 16 proteins, including those with lower molecular weights, showed a statistically lower presence in samples with a PMI >13 years compared to the samples with PMIs <12 years. In addition, the proportion of acidic and neutral amino acids increased with the PMI, while that of basic amino acids decreased. Both studies were assigned with a moderate risk of bias in part because of outcome-oriented sample splitting of compared groups and evidence of repeated use of samples.

Zissler et al. 2018 [30] used SDS-PAGE based western blotting to analyze the degradation of desmin, vinculin and tropomyosin in rat hindlimb muscle. A total of 20 rats were equally divided into groups of $n = 4$. After different pretreatment (injury, induction of atrophy by cervical dislocation) the 16 animals were killed and stored for up to 4 days postmortem at constant temperature of 20 °C. The remaining animals served as controls to document state at 0 hpm. Desmin and (meta-)vinculin were found to degrade in a time-dependent manner, the native desmin band being lost at 2 dpm, and both proteins giving rise to several degradation products. By contrast, tropomyosin remained stable over the investigated 4 day PMI. In addition, results demonstrated an effect of altered antemortem metabolism (resulting from injury and atrophy) on postmortem protein decomposition. The study was assigned with a low risk of bias.

Jellinghaus et al. published two studies on postmortem degradation of collagen. The first study, of 2018, undertook a validation following to the work of Boaks et al. [10], determining collagen to non-collagen (Co/NCo) ratios after differential staining with Sirius Red/Fast Green in 16 porcine bones [31]. Specimens were buried in plastic boxes and stored under monitored conditions for 3 month and sampled at 3 times. As outcomes of eluate photometry after destaining differed markedly

from those of Boaks et al. [10], the authors tested their results with a new second method. They examined sections of the stained bones under a stereomicroscope and calculated Co/NCo ratios from digital imaging data. The authors found a first positive, later negative correlation of the Co/NCo ratio with the PMI, showing a significant increase until 56 dpm and a subsequent significant reduction over the rest of the investigated 3 month period. In addition, the study tested how the presence of microorganisms instilled by hay infusions influenced bone collagen degradation, but found no significant effects. Although the study is generally well designed, the lack of reported effort to reduce bias in selecting a homogenous animal population leads to a moderate risk of bias.

In the second study, published in 2019 [32], the authors used human bones. 37 femur samples were collected during exhumations (PMI 21-48 years) and autopsies, and samples of 11 femoral bones (PMI 135-153 years) were received from archaeological cases. Using the methods of the first study, the authors report an ascending trend of the Co/nCo ratio if measured photometrically, but a decreasing trend if measurement is made with stereomicroscopic image analysis. If sorted by sex, the concentration of the Co/nCo ratio decreased in males ($R= -0.62$), but increased in females ($R = 0.24$). Inaccuracies in describing the study design/outcome, as well as a lack in reporting sampling details, influencing factors and case data resulted in a high risk of bias rating.

Alibegović et al (2019) investigated cartilage degradation in bone samples of human donors stored for up to 36 dpm at 11 °C and 35 °C. Intensities of different classical histological collagen and proteoglycan stainings were assessed by blinded observers using a grading scale. The authors found a significant reduction in staining intensity at day 36 postmortem at both temperatures, compared to the baseline samples. They also report that postmortem time had a larger (significant) effect than temperature. Due to a small sample size, the in other respects well designed study was associated with a high risk of bias.

Cho and Eom (2019) [33] used western blots and IHC to test whether the multi-ligand receptor for advanced glycation end products (RAGE) could serve as a biomarker for PMI estimation in drowning cases. Lung tissue of drowned rats was dissected at 7 time points postmortem (1 to 7 dpm, 5 animals per time point) after controlled storage of the cadavers in sea water (15 ± 5 °C). Non-drowned rats immersed in sea water and untreated rats served as controls. Western blots showed that RAGE protein expression decreased significantly over the postmortem submersion interval. Similarly, the additional IHC analysis revealed a gradual decrease in staining intensity of RAGE. Western blot analysis was associated with low risk of bias, the IHC investigation with a moderate risk of bias (assessment of staining intensity insufficiently described).

Choi et al. (2019) [34] presented a method to discover new protein markers for PMI estimation by mass spectrometry. Muscle tissue (M. vastus lateralis) samples of rats and mice ($n = 2$ per time point each) collected at 5 time points postmortem (PMI 0–96 hpm, stored at 20°C) were analyzed for the degradation of glycerinaldehyd-3-phosphat-dehydrogenase (GAPDH) and eukaryotic translation elongation factor 1 alpha 2 (eEF1A2). Results showed that both proteins degrade consistently over the PMI in both animal models. The usefulness of these proteins as PMI estimation markers was additionally validated by western blot analysis in rats ($n = 5$ each per time point) and humans (3 samples of 3 autopsy cases). In a standardized degradation model, degradation patterns of GAPDH and eEF1A2 in the rats were also compared to those of tropomyosin, desmin and vinculin. Tropomyosin and GAPDH bands were present at all time points, whereas the intensity of the GAPDH band decreased significantly over the 96 hpm interval. eEF1A2 was completely degraded by 48 hpm, and native desmin bands were lost at 96 hpm. Vinculin bands also decreased in intensity over the investigated PMI. The two latter proteins produced several degradation products. To assess risk of bias, the study was separated according to methods and species. An appropriate number of rats was used for western blot analysis (low risk of bias); by contrast, only low numbers of samples were analyzed by mass spectrometry (rats), and by western blotting (humans), resulting in the assignment of a high risk of bias.

Da Fonseca et al. (2019) [35] used enzyme activity assays to determine the postmortem activity of acetylcholinesterase (AChE), Na+/K+-ATPase and gluathione-S-transferase (GST) in mice. Whole brain and samples of liver, kidney and gastrocnemius muscle were harvested from 6 to 7 animals

(stored at 22 ± 2 °C) at 4 time points from 0-48 hpm. Results showed a significant reduction of Na+/K+-ATPase activity over the investigated PMI in the brain, and a significant increase of GST and Na+/K+-ATPase in the kidney. Varying levels of AChE and GST were found in brain and liver. No time-dependent changes were detected in the activity levels of GST in brain and muscle. The study was associated with low risk of bias.

References

1. Wehner, F.; Wehner, H.-D.; Schieffer, M.C.; Subke, J. Delimitation of the time of death by immunohistochemical detection of insulin in pancreatic β-cells. *Forensic Sci. Int.* **1999**, *105*, 161–169, doi:10.1016/S0379-0738(99)00124-3.
2. Wehner, F.; Wehner, H.-D.; Subke, J. Delimitation of the time of death by immunohistochemical detection of glucagon in pancreatic α-cells. *Forensic Sci. Int.* **2001**, *124*, 192–199, doi:10.1016/S0379-0738(01)00608-9.
3. Wehner, F.; Wehner, H.-D.; Schieffer, M.C.; Subke, J. Delimitation of the time of death by immunohistochemical detection of thyroglobulin. *Forensic Sci. Int.* **2000**, *110*, 199–206, doi:10.1016/S0379-0738(00)00177-8.
4. Wehner, F.; Wehner, H.-D.; Subke, J. Delimitation of the time of death by immunohistochemical detection of calcitonin. *Forensic Sci. Int.* **2001**, *122*, 89–94, doi:10.1016/S0379-0738(01)00467-4.
5. Thaik-Oo, M.; Tanaka, E.; Tsuchiya, T.; Kominato, Y.; Honda, K.; Yamazaki, K.; Misawa, S. Estimation of postmortem interval from hypoxic inducible levels of vascular endothelial growth factor. *J. Forensic Sci.* **2002**, *47*, 186–189.
6. Sabucedo, A.J.; Furton, K.G. Estimation of postmortem interval using the protein marker cardiac Troponin I. *Forensic Sci. Int.* **2003**, *134*, 11–16.
7. Kang, S.; Kassam, N.; Gauthier, M.L.; O'Day, D.H. Post-mortem changes in calmodulin binding proteins in muscle and lung. *Forensic Sci. Int.* **2003**, *131*, 140–147, doi:10.1016/S0379-0738(02)00426-7.
8. Poloz, Y.O.; O'Day, D.H. Determining time of death: Temperature-dependent postmortem changes in calcineurin A, MARCKS, CaMKII, and protein phosphatase 2A in mouse. *Int J. Leg. Med.* **2009**, *123*, 305–314, doi:10.1007/s00414-009-0343-x.
9. Wehner, F.; Steinriede, A.; Martin, D.; Wehner, H.-D. Two-tailed delimitation of the time of death by immunohistochemical detection of somatostatin and GFAP. *Sci Med. Pathol* **2006**, *2*, 241–247, doi:10.1385/FSMP:2:4:241.
10. Boaks, A.; Siwek, D.; Mortazavi, F. The temporal degradation of bone collagen: A histochemical approach. *Forensic Sci. Int.* **2014**, *240*, 104–110, doi:10.1016/j.forsciint.2014.04.008.
11. Kumar, S.; Ali, W.; Singh, U.S.; Kumar, A.; Bhattacharya, S.; Verma, A.K.; Rupani, R. Temperature-Dependent Postmortem Changes in Human Cardiac Troponin-T (cTnT): An Approach in Estimation of Time Since Death. *J. Forensic Sci* **2016**, *61*, S241–S245, doi:10.1111/1556-4029.12928.
12. Kumar, S.; Ali, W.; Singh, U.S.; Kumar, A.; Bhattacharya, S.; Verma, A.K. The effect of elapsed time on the cardiac Troponin-T (cTnT) proteolysis in case of death due to burn: A study to evaluate the potential forensic use of cTnT to determine the postmortem interval. *Sci. Justice* **2015**, *55*, 189–194, doi:10.1016/j.scijus.2014.12.006.
13. Kumar, S.; Ali, W.; Bhattacharya, S.; Singh, U.S.; Kumar, A.; Verma, A.K. The effect of elapsed time on cardiac troponin-T (cTnT) degradation and its relation to postmortem interval in cases of electrocution. *J. Forensic Leg. Med.* **2015**, *34*, 45–49, doi:10.1016/j.jflm.2015.05.009.
14. Kumar, S.; Ali, W.; Singh, U.S.; Verma, A.K.; Bhattacharya, S.; Kumar, A.; Singh, R.; Rupani, R. Time-dependent Degradation Pattern of Cardiac Troponin T in Cases of Death by Burn. *West. Indian Med. J.* **2015**, *64*, 226–229, doi:10.7727/wimj.2014.232.
15. Bolton, S.N.; Whitehead, M.P.; Dudhia, J.; Baldwin, T.C.; Sutton, R. Investigating the Postmortem Molecular Biology of Cartilage and its Potential Forensic Applications. *J. Forensic Sci.* **2015**, *60*, 1061–1067, doi:10.1111/1556-4029.12764.
16. Abo El-Noor, M.M.; Elhosary, N.M.; Khedr, N.F.; El-Desouky, K.I. Estimation of Early Postmortem Interval Through Biochemical and Pathological Changes in Rat Heart and Kidney. *Am. J. Forensic Med. Pathol* **2016**, *37*, 40–46, doi:10.1097/PAF.0000000000000214.
17. Foditsch, E.E.; Saenger, A.M.; Monticelli, F.C. Skeletal muscle proteins: A new approach to delimitate the time since death. *Int. J. Leg. Med.* **2016**, *130*, 433–440, doi:10.1007/s00414-015-1204-4.

18. Pittner, S.; Monticelli, F.C.; Pfisterer, A.; Zissler, A.; Sänger, A.M.; Stoiber, W.; Steinbacher, P. Postmortem degradation of skeletal muscle proteins: A novel approach to determine the time since death. *Int. J. Leg. Med.* **2016**, *130*, 421–431, doi:10.1007/s00414-015-1210-6.
19. Pittner, S.; Ehrenfellner, B.; Monticelli, F.C.; Zissler, A.; Sänger, A.M.; Stoiber, W.; Steinbacher, P. Postmortem muscle protein degradation in humans as a tool for PMI delimitation. *Int. J. Leg. Med.* **2016**, doi:10.1007/s00414-016-1349-9.
20. Pittner, S.; Ehrenfellner, B.; Zissler, A.; Racher, V.; Trutschnig, W.; Bathke, A.C.; Sänger, A.M.; Stoiber, W.; Steinbacher, P.; Monticelli, F.C. First application of a protein-based approach for time since death estimation. *Int J. Leg. Med.* **2017**, *131*, 479–483, doi:10.1007/s00414-016-1459-4.
21. Li, C.; Wang, Q.; Zhang, Y.; Lin, H.; Zhang, J.; Huang, P.; Wang, Z. Research progress in the estimation of the postmortem interval by Chinese forensic scholars. *Forensic Sci Res.* **2016**, *1*, 3–13, doi:10.1080/20961790.2016.1229377.
22. Lee, D.-G.; Yang, K.E.; Hwang, J.W.; Kang, H.-S.; Lee, S.-Y.; Choi, S.; Shin, J.; Jang, I.-S.; An, H.J.; Chung, H.; et al. Degradation of Kidney and Psoas Muscle Proteins as Indicators of Post-Mortem Interval in a Rat Model, with Use of Lateral Flow Technology. *Plos One* **2016**, *11*, e0160557, doi:10.1371/journal.pone.0160557.
23. Ortmann, J.; Doberentz, E.; Madea, B. Immunohistochemical methods as an aid in estimating the time since death. *Forensic Sci. Int.* **2017**, *273*, 71–79, doi:10.1016/j.forsciint.2017.02.004.
24. Kwak, J.-H.; Kim, H.K.; Kim, K.; Noh, B.R.; Cheon, H.I.; Yeo, M.; Shakya, R.; Shrestha, S.A.; Kim, D.; Choe, S.; et al. Proteomic Evaluation of Biomarkers to Determine the Postmortem Interval. *Anal. Lett.* **2017**, *50*, 207–218, doi:10.1080/00032719.2016.1172080.
25. Li, C.; Li, Z.; Tuo, Y.; Ma, D.; Shi, Y.; Zhang, Q.; Zhuo, X.; Deng, K.; Chen, Y.; Wang, Z.; et al. MALDI-TOF MS as a Novel Tool for the Estimation of Postmortem Interval in Liver Tissue Samples. *Sci. Rep.* **2017**, *7*, 4887, doi:10.1038/s41598-017-05216-0.
26. Li, C.; Ma, D.; Deng, K.; Chen, Y.; Huang, P.; Wang, Z. Application of MALDI-TOF MS for Estimating the Postmortem Interval in Rat Muscle Samples. *J. Forensic Sci.* **2017**, *62*, 1345–1350, doi:10.1111/1556-4029.13413.
27. Pérez-Martínez, C.; Pérez-Cárceles, M.D.; Legaz, I.; Prieto-Bonete, G.; Luna, A. Quantification of nitrogenous bases, DNA and Collagen type I for the estimation of the postmortem interval in bone remains. *Forensic Sci. Int.* **2017**, *281*, 106–112, doi:10.1016/j.forsciint.2017.10.039.
28. Procopio, N.; Williams, A.; Chamberlain, A.T.; Buckley, M. Forensic proteomics for the evaluation of the post-mortem decay in bones. *J. Proteom.* **2018**, *177*, 21–30, doi:10.1016/j.jprot.2018.01.016.
29. Prieto-Bonete, G.; Pérez-Cárceles, M.D.; Maurandi-López, A.; Pérez-Martínez, C.; Luna, A. Association between protein profile and postmortem interval in human bone remains. *J. Proteom.* **2018**, doi:10.1016/j.jprot.2018.08.008.
30. Zissler, A.; Ehrenfellner, B.; Foditsch, E.E.; Monticelli, F.C.; Pittner, S. Does altered protein metabolism interfere with postmortem degradation analysis for PMI estimation? *Int. J. Leg. Med.* **2018**, doi:10.1007/s00414-018-1814-8.
31. Jellinghaus, K.; Hachmann, C.; Höland, K.; Bohnert, M.; Wittwer-Backofen, U. Collagen degradation as a possibility to determine the post-mortem interval (PMI) of animal bones: A validation study referring to an original study of Boaks et al. (2014). *Int. J. Leg. Med.* **2018**, *132*, 753–763, doi:10.1007/s00414-017-1747-7.
32. Jellinghaus, K.; Urban, P.K.; Hachmann, C.; Bohnert, M.; Hotz, G.; Rosendahl, W.; Wittwer-Backofen, U. Collagen degradation as a possibility to determine the post-mortem interval (PMI) of human bones in a forensic context—A survey. *Leg. Med.* **2019**, *36*, 96–102, doi:10.1016/j.legalmed.2018.11.009.
33. Cho, H.-W.; Eom, Y.-B. Potential Forensic Application of Receptor for Advanced Glycation End Products (RAGE) as a Novel Biomarker for Estimating Postmortem Interval. *J. Forensic Sci.* **2019**, *64*, 1878–1883, doi:10.1111/1556-4029.14063.
34. Choi, K.-M.; Zissler, A.; Kim, E.; Ehrenfellner, B.; Cho, E.; Lee, S.; Steinbacher, P.; Yun, K.N.; Shin, J.H.; Kim, J.Y.; et al. Postmortem proteomics to discover biomarkers for forensic PMI estimation. *Int J. Leg. Med.* **2019**, *133*, 899–908, doi:10.1007/s00414-019-02011-6.
35. da Fonseca, C.A.R.; Paltian, J.; dos Reis, A.S.; Bortolatto, C.F.; Wilhelm, E.A.; Luchese, C. Na+/K+-ATPase, acetylcholinesterase and glutathione S-transferase activities as new markers of postmortem interval in Swiss mice. *Leg. Med.* **2019**, *36*, 67–72, doi:10.1016/j.legalmed.2018.11.003.
