# Supplementary material for: Postmortem Protein Degradation as a Tool to Estimate the PMI: A Systematic Review
Source: Diagnostics (Basel). 2020 Nov 26;10(12):1014. doi: 10.3390/diagnostics10121014 (PMC7760775; doi:10.3390/diagnostics10121014)
Supplement: Supplementary file 1 [file diagnostics-10-01014-s001.zip › File S2.pdf]

RISK OF BIAS JUDGEMENT

1.1	1.2.1	1.2.2	risk of bias
Y/PY	Y/PY	Y/PY	low
		N/PN/NI	moderate
	N/PN/NI		high
N/PN/NI			high

2.1	2.2	2.3	2.4	2.5	risk of bias
Y	Y	Y	Y	Y	low
				N	moderate
			N	Y	moderate
				N	high
		N	Y	Y	moderate
				N	high
			N		high
	N	Y	Y	Y	moderate
				N	high
			N		high
		N			high
N	Y	Y	Y	Y	moderate
				N	high
			N		high
		N			high
	N				high

3.1	3.2.1	3.2.2	3.2.3	risk of bias
Y/PY				low
N/PN/NI	Y/PY	Y	Y	low
			N/NI	moderate
		N		high
	N/PN/NI	Y	Y	moderate
			N/NI	high
		N		high

4.1	4.2	risk of bias
Y/PY/NI	Y/PY	high
	N/PN	moderate
N/PN		low

5.1	5.2	risk of bias
Y/PY/NI	Y/PY/NI	high
	N/PN	moderate
N/PN	Y/PY/NI	moderate
	N/PN	low

6.1	6.2.1	6.2.2	6.3	risk of bias
Y/PY	Y/PY	Y/PY		high
		N/PN	Y/PY	high
			N/PN	moderate
	N/PN		Y/PY	high
			N/PN	moderate
N/PN				low

OVERALL RISK OF BIAS JUDGEMENT

<table>
<tr><th>Bias arising from study design</th><th>Bias due to inaccuracy in reporting the method</th><th>Bias due to missing outcome data</th><th>Bias in measurement of the outcome</th><th>Bias in selection of the reported result</th><th>Bias arising from multiple use of data</th><th>Overall risk of bias</th></tr>
<tr><td rowspan="63">low</td><td rowspan="31">low</td><td rowspan="15">low</td><td rowspan="7">low</td><td rowspan="3">low</td><td>low</td><td>low</td></tr>
<tr><td>moderate</td><td>moderate</td></tr>
<tr><td>high</td><td>high</td></tr>
<tr><td rowspan="3">moderate</td><td>low</td><td>moderate</td></tr>
<tr><td>moderate</td><td>moderate</td></tr>
<tr><td>high</td><td>high</td></tr>
<tr><td colspan="2">high</td><td>high</td></tr>
<tr><td rowspan="7">moderate</td><td rowspan="3">low</td><td>low</td><td>moderate</td></tr>
<tr><td>moderate</td><td>moderate</td></tr>
<tr><td>high</td><td>high</td></tr>
<tr><td rowspan="3">moderate</td><td>low</td><td>moderate</td></tr>
<tr><td>moderate</td><td>moderate</td></tr>
<tr><td>high</td><td>high</td></tr>
<tr><td colspan="2">high</td><td>high</td></tr>
<tr><td colspan="3">high</td><td>high</td></tr>
<tr><td rowspan="15">moderate</td><td rowspan="7">low</td><td rowspan="3">low</td><td>low</td><td>moderate</td></tr>
<tr><td>moderate</td><td>moderate</td></tr>
<tr><td>high</td><td>high</td></tr>
<tr><td rowspan="3">moderate</td><td>low</td><td>moderate</td></tr>
<tr><td>moderate</td><td>moderate</td></tr>
<tr><td>high</td><td>high</td></tr>
<tr><td colspan="2">high</td><td>high</td></tr>
<tr><td rowspan="7">moderate</td><td rowspan="3">low</td><td>low</td><td>moderate</td></tr>
<tr><td>moderate</td><td>moderate</td></tr>
<tr><td>high</td><td>high</td></tr>
<tr><td rowspan="3">moderate</td><td>low</td><td>moderate</td></tr>
<tr><td>moderate</td><td>moderate</td></tr>
<tr><td>high</td><td>high</td></tr>
<tr><td colspan="2">high</td><td>high</td></tr>
<tr><td colspan="3">high</td><td>high</td></tr>
<tr><td colspan="4">high</td><td>high</td></tr>
<tr><td rowspan="31">moderate</td><td rowspan="15">low</td><td rowspan="7">low</td><td rowspan="3">low</td><td>low</td><td>moderate</td></tr>
<tr><td>moderate</td><td>moderate</td></tr>
<tr><td>high</td><td>high</td></tr>
<tr><td rowspan="3">moderate</td><td>low</td><td>moderate</td></tr>
<tr><td>moderate</td><td>moderate</td></tr>
<tr><td>high</td><td>high</td></tr>
<tr><td colspan="2">high</td><td>high</td></tr>
<tr><td rowspan="7">moderate</td><td rowspan="3">low</td><td>low</td><td>moderate</td></tr>
<tr><td>moderate</td><td>moderate</td></tr>
<tr><td>high</td><td>high</td></tr>
<tr><td rowspan="3">moderate</td><td>low</td><td>moderate</td></tr>
<tr><td>moderate</td><td>moderate</td></tr>
<tr><td>high</td><td>high</td></tr>
<tr><td colspan="2">high</td><td>high</td></tr>
<tr><td colspan="3">high</td><td>high</td></tr>
<tr><td rowspan="15">moderate</td><td rowspan="7">low</td><td rowspan="3">low</td><td>low</td><td>moderate</td></tr>
<tr><td>moderate</td><td>moderate</td></tr>
<tr><td>high</td><td>high</td></tr>
<tr><td rowspan="3">moderate</td><td>low</td><td>moderate</td></tr>
<tr><td>moderate</td><td>moderate</td></tr>
<tr><td>high</td><td>high</td></tr>
<tr><td colspan="2">high</td><td>high</td></tr>
<tr><td rowspan="7">moderate</td><td rowspan="3">low</td><td>low</td><td>moderate</td></tr>
<tr><td>moderate</td><td>moderate</td></tr>
<tr><td>high</td><td>high</td></tr>
<tr><td rowspan="3">moderate</td><td>low</td><td>moderate</td></tr>
<tr><td>moderate</td><td>moderate</td></tr>
<tr><td>high</td><td>high</td></tr>
<tr><td colspan="2">high</td><td>high</td></tr>
<tr><td colspan="3">high</td><td>high</td></tr>
<tr><td colspan="4">high</td><td>high</td></tr>
<tr><td colspan="5">high</td><td>high</td></tr>
</table>

OVERALL RISK OF BIAS JUDGEMENT

Bias arising from study design	Bias due to inaccuracy in reporting the method	Bias due to missing outcome data	Bias in measurement of the outcome	Bias in selection of the reported result	Bias arising from multiple use of data	Overall risk of bias
moderate	low	low	low	low	low	**moderate**
					moderate	**moderate**
					high	**high**
				moderate	low	**moderate**
					moderate	**moderate**
					high	**high**
				high		**high**
			moderate	low	low	**moderate**
					moderate	**moderate**
					high	**high**
				moderate	low	**moderate**
					moderate	**moderate**
					high	**high**
				high		**high**
			high			**high**
		moderate	low	low	low	**moderate**
					moderate	**moderate**
					high	**high**
				moderate	low	**moderate**
					moderate	**moderate**
					high	**high**
				high		**high**
			moderate	low	low	**moderate**
					moderate	**moderate**
					high	**high**
				moderate	low	**moderate**
					moderate	**moderate**
					high	**high**
				high		**high**
			high			**high**
		high				**high**
	moderate	low	low	low	low	**moderate**
					moderate	**moderate**
					high	**high**
				moderate	low	**moderate**
					moderate	**moderate**
					high	**high**
				high		**high**
			moderate	low	low	**moderate**
					moderate	**moderate**
					high	**high**
				moderate	low	**moderate**
					moderate	**moderate**
					high	**high**
				high		**high**
			high			**high**
		moderate	low	low	low	**moderate**
					moderate	**moderate**
					high	**high**
				moderate	low	**moderate**
					moderate	**moderate**
					high	**high**
				high		**high**
			moderate	low	low	**moderate**
					moderate	**moderate**
					high	**high**
				moderate	low	**moderate**
					moderate	**moderate**
					high	**high**
				high		**high**
			high			**high**
		high				**high**
	high					**high**
high						**high**
