# Supplementary material for: Postmortem Protein Degradation as a Tool to Estimate the PMI: A Systematic Review
Source: Diagnostics (Basel). 2020 Nov 26;10(12):1014. doi: 10.3390/diagnostics10121014 (PMC7760775; doi:10.3390/diagnostics10121014)
Supplement: Supplementary file 1 [file diagnostics-10-01014-s001.zip › Table S1.pdf]

protein	abbreviation	method	tissue	reference
A disintegrin and metalloproteinase with thrombospondin motifs 17	ADAMTS17	mass spectrometry	bone	Prieto-Bonete et al. 2018 [25]
A disintegrin and metalloproteinase with thrombospondin motifs 18	ADAMTS18	mass spectrometry	bone	Prieto-Bonete et al. 2018 [25]
acetyl-coenzyme A acyltransferase 2		2D-PAGE	liver	Kwak et al. 2017 [52]
acetylcolagenesterase	AChE	enzyme activity assay	brain, liver, skeletal muscle, kidney	da Fonseca et al. 2019 [60]
actin		western blot	heart, liver, spleen, lung, kidney, brain, skeletal muscle	Li et al. 2016 [27]
actin		immunohistochemistry	heart, skeletal muscle	Li et al. 2016 [27]
adenosine kinase		2D-PAGE	liver	Kwak et al. 2017 [52]
adenosine triphosphate synthase, H+transporting, mitochondrial F1 complex, alpha subunit 1, cardiac muscle		2D-PAGE	heart	Kwak et al. 2017 [52]
aggrecan		western blot	cartilage	Bolton et al. 2015 [56]
albumin	ALBU	mass spectrometry	bone	Procopio et al. 2018 [59]
aldehyde dehydrogenase, mitochondrial precursor		2D-PAGE	liver	Kwak et al. 2017 [52]
aldolase A		2D-PAGE	heart	Kwak et al. 2017 [52]
alpha 1 type XXIV collagen precursor	COL24A1	mass spectrometry	bone	Prieto-Bonete et al. 2018 [25]
alpha actinin		western blot	skeletal muscle	Foditsch et al. 2016 [57]
alpha actinin		western blot	skeletal muscle	Pittner et al. 2016 [23]
alpha B-crystalline		2D-PAGE	heart	Kwak et al. 2017 [52]
alpha enolase		2D-PAGE	liver	Kwak et al. 2017 [52]
alpha-2-HS-glycoprotein	AHSG	mass spectrometry	bone	Prieto-Bonete et al. 2018 [25]
AMP-activated protein kinase α	AMPK-α	western blot	skeletal muscle	Lee et al. 2016 [50]
amphiregulin preproprotein	AREG	mass spectrometry	bone	Prieto-Bonete et al. 2018 [25]
annexin A1	ANXA1	mass spectrometry	bone	Procopio et al. 2018 [59]
annexin II receptor	ANXA2R	mass spectrometry	bone	Prieto-Bonete et al. 2018 [25]
apolipoprotein A1	APOA1	mass spectrometry	bone	Procopio et al. 2018 [59]
apolipoprotein C3	APOC3	mass spectrometry	bone	Procopio et al. 2018 [59]
arginase-1		2D-PAGE	liver	Kwak et al. 2017 [52]
beta catenin		western blot	skeletal muscle	Lee et al. 2016 [50]
beta enolase	ENOB	mass spectrometry	bone	Procopio et al. 2018 [59]
beta-catenin 1	CTNNB	mass spectrometry	bone	Prieto-Bonete et al. 2018 [25]
bone morphogenetic protein 5 preproprotein	BMP5	mass spectrometry	bone	Prieto-Bonete et al. 2018 [25]
c alpha-1(II) chain	COL2A1	mass spectrometry	bone	Prieto-Bonete et al. 2018 [25]
Ca2+/calmodulin-dependent protein kinase II	CaMKII	western blot	lung, skeletal muscle	Kang et al. 2003 [48]
Ca2+/calmodulin-dependent protein kinase II	CaMKII	western blot	lung, skeletal muscle	Poloz and O'Day 2009 [24]
cadherin-11	CDH11	mass spectrometry	bone	Prieto-Bonete et al. 2018 [25]
calcineurin A	CnA	western blot	lung, skeletal muscle	Kang et al. 2003 [48]
calcineurin A	CnA	western blot	lung, skeletal muscle	Poloz and O'Day 2009 [24]
calcitonin		immunohistochemistry	thyroid gland	Ortmann et al. 2017 [43]
calcitonin		immunohistochemistry	thyroid gland	Wehner et al. 2001 [32]
calpain 1 (µ-calpain)		western blot	skeletal muscle	Foditsch et al. 2016 [57]
calpain 1 (µ-calpain)		casein zymography	skeletal muscle	Pittner et al. 2016 [23]
calpain 1 (µ-calpain)		casein zymography	skeletal muscle	Pittner et al. 2016 [40]
calpain 1 (µ-calpain)		western blot	skeletal muscle	Pittner et al. 2017 [41]
calpain 2 (m-calpain)		casein zymography	skeletal muscle	Pittner et al. 2016 [23]
calpain 2 (m-calpain)		casein zymography	skeletal muscle	Pittner et al. 2016 [40]
calpain 2 (m-calpain)		casein zymography	skeletal muscle	Pittner et al. 2017 [41]
calsequestrin 1		western blot	skeletal muscle	Foditsch et al. 2016 [57]
cardiac myosin heavy chain 5		2D-PAGE	heart	Kwak et al. 2017 [52]
cardiac troponin I	cTnI	mass spectrometry	heart	Sabucedo and Furton 2003 [35]
cardiac troponin T		western blot	skeletal muscle	Foditsch et al. 2016 [57]
cardiac troponin T		western blot	heart	Kumar et al. 2015 [36]
cardiac troponin T		western blot	heart	Kumar et al. 2015 [37]
cardiac troponin T		western blot	heart	Kumar et al. 2015 [38]
cardiac troponin T		western blot	heart	Kumar et al. 2016 [39]
cardiac troponin T	cTnT	western blot	skeletal muscle	Pittner et al. 2016 [23]
cardiac troponin T	cTnT	western blot	skeletal muscle	Pittner et al. 2016 [40]
cardiac troponin T	cTnT	western blot	skeletal muscle	Pittner et al. 2017 [41]
cartilage intermediate layer protein 1	CILP	mass spectrometry	bone	Prieto-Bonete et al. 2018 [25]
cartilage oligomerix matrix protein	COMP	mass spectrometry	bone	Prieto-Bonete et al. 2018 [25]
caspase 3		immunohistochemistry, western blot	kidney, skeletal muscle	Lee et al. 2016 [50]
catalase	CAT	enzyme activity assay	heart, kidney	Abo El-Noor et al. 2016 [49]
collagen		histology	cartilage	Alibegović et al. 2019 [46]
collagen		histology/photometry	bone	Boaks et al. 2014 [55]
collagen		histology, histology/photometry	bone	Jellinghaus et al. 2018 [58]
collagen		histology, histology/photometry	bone	Jellinghaus et al. 2019 [45]
collagen alpha-1 (V)	COL5A1	mass spectrometry	bone	Prieto-Bonete et al. 2018 [25]
collagen alpha-1(I) chain	COL1A1	mass spectrometry	bone	Prieto-Bonete et al. 2018 [25]
collagen alpha-1(III) chain	COL3A1	mass spectrometry	bone	Prieto-Bonete et al. 2018 [25]
collagen alpha-1(IX) chain	COL9A1	mass spectrometry	bone	Prieto-Bonete et al. 2018 [25]
collagen alpha-1(VII) chain precursor	COL7A1	mass spectrometry	bone	Prieto-Bonete et al. 2018 [25]
collagen alpha-1(X)	COL10A1	mass spectrometry	bone	Prieto-Bonete et al. 2018 [25]
collagen alpha-1(XI) chain	COL11A1	mass spectrometry	bone	Prieto-Bonete et al. 2018 [25]
collagen alpha-1(XXVII) chain preproprotein	COL27A1	mass spectrometry	bone	Prieto-Bonete et al. 2018 [25]
collagen alpha-2(I)	COL1A2	mass spectrometry	bone	Prieto-Bonete et al. 2018 [25]
collagen alpha-2(IV) chain preproprotein	COL4A2	mass spectrometry	bone	Prieto-Bonete et al. 2018 [25]
collagen alpha-2(IX) chain,	COL9A2	mass spectrometry	bone	Prieto-Bonete et al. 2018 [25]
collagen alpha-2(V) chain	COL5A2	mass spectrometry	bone	Prieto-Bonete et al. 2018 [25]
collagen alpha-2(XI) chain	COL11A2	mass spectrometry	bone	Prieto-Bonete et al. 2018 [25]
collagen type 1		mass spectrometry	bone	Pérez-Martínez e al. 2017 [42]
collagen type 5	CO5A2 (vermutl falsch abgek)	mass spectrometry	bone	Procopio et al. 2018 [59]
creatine kinase M type	KCRM	mass spectrometry	bone	Procopio et al. 2018 [59]
creatine kinase, muscle		2D-PAGE	heart	Kwak et al. 2017 [52]
CST complex subunit CTC1	CTC1	mass spectrometry	bone	Prieto-Bonete et al. 2018 [25]
desmin	DES	western blot	skeletal muscle	Choi et al. 2019 [47]
desmin	DE-R-11	western blot	skeletal muscle	Foditsch et al. 2016 [57]
desmin		2D- PAGE	heart	Kwak et al. 2017 [52]
desmin	DES	western blot	skeletal muscle	Pittner et al. 2016 [23]
desmin	DES	western blot	skeletal muscle	Pittner et al. 2016 [40]
desmin	DES	western blot	skeletal muscle	Pittner et al. 2017 [41]
desmin	DES	western blot	skeletal muscle	Zissler et al. 2018 [51]
ectonucleotide pyrophosphatase/phosphodiesterase family member 1	ENPP1	mass spectrometry	bone	Prieto-Bonete et al. 2018 [25]
eucaryotic translation elongation factor 1 alpha 2	eEF1A2	mass spectrometry, western blot	skeletal muscle	Choi et al. 2019 [47]
fibrillin-1	FBN1	mass spectrometry	bone	Prieto-Bonete et al. 2018 [25]
fibroblast growth factor receptor 1	FGFR1	mass spectrometry	bone	Prieto-Bonete et al. 2018 [25]
fructose-bisphosphate aldolase A	ALDOA	mass spectrometry	bone	Procopio et al. 2018 [59]
glial fibrillary acidic protein	GFAP	immunohistochemistry	brain, pankreas	Wehner et al. 2006 [29]

protein	abbreviation	method	tissue	reference
glial fibrillary acidic protein	GFAP	immunohistochemistry	brain, pankreas	Wehner et al. 2006 [29]
glucagon		immunohistochemistry	pancreas	Ortmann et al. 2017 [43]
glucagon		immunohistochemistry	pancreas	Wehner et al. 2001 [30]
glutathione S transferase	GST	enzyme activity assay	heart, kidney	Abo El-Noor et al. 2016 [49]
glutathione S-transferase alpha-5		2D-PAGE	liver	Kwak et al. 2017 [52]
glutathione S-transferase mu 2		2D-PAGE	liver	Kwak et al. 2017 [52]
glutathione synthetase		2D-PAGE	liver	Kwak et al. 2017 [52]
glutathione-S-transferase	GST	enzyme activity assay	brain, liver, skeletal muscle, kidney	da Fonseca et al. 2019 [60]
gluthatione reductase	GR	enzyme activity assay	heart, kidney	Abo El-Noor et al. 2016 [49]
glycerinaldehyd-3-phosphat-Dehydrogenase	GAPDH	mass spectrometry, western blot	skeletal muscle	Choi et al. 2019 [47]
glycerinaldehyd-3-phosphat-Dehydrogenase	GAPDH	lateral flow assay, western blot	kidney, skeletal muscle	Lee et al. 2016 [50]
glycogen synthase	GS	immunohistochemistry, western blot	kidney, skeletal muscle	Lee et al. 2016 [50]
glycogen synthase kinase-3 β	GSK-3β	western blot	kidney	Lee et al. 2016 [50]
haemoglobulin beta	HBB	mass spectrometry	bone	Procopio et al. 2018 [59]
haeoglobulin alpha	HBA	mass spectrometry	bone	Procopio et al. 2018 [59]
haptoglobin	HPT	mass spectrometry	bone	Procopio et al. 2018 [59]
histone H2A deubiquitinase MYSM1 MYSM1		mass spectrometry	bone	Prieto-Bonete et al. 2018 [25]
homeobox protein DLX-5	DLX5	mass spectrometry	bone	Prieto-Bonete et al. 2018 [25]
hydroxyacylglutathione hydrolase		2D-PAGE	liver	Kwak et al. 2017 [52]
inducible nitric oxide synthase	iNOS	western blot	lung, skeletal muscle	Kang et al. 2003 [48]
insulin		immunohistochemistry	pancreas	Ortmann et al. 2017 [43]
insulin		immunohistochemistry	pancreas	Wehner et al. 1999 [31]
insulin-induced gene 2 protein	INSIG2	mass spectrometry	bone	Prieto-Bonete et al. 2018 [25]
lactate dehydrogenase B		2D-PAGE	heart	Kwak et al. 2017 [52]
lactotransferrin	TRFL	mass spectrometry	bone	Procopio et al. 2018 [59]
laminin		western blot	skeletal muscle	Foditsch et al. 2016 [57]
laminin subunit alpha-2	LAMA2	mass spectrometry	bone	Prieto-Bonete et al. 2018 [25]
malate dehydrogenase 1, nicotinamide adenine dinucleotide (soluble)		2D-PAGE	heart	Kwak et al. 2017 [52]
matrix Gla protein	MGP	mass spectrometry	bone	Procopio et al. 2018 [59]
mediator of RNA polymerase II transcription subunit 12	MED12	mass spectrometry	bone	Prieto-Bonete et al. 2018 [25]
mucin-15	MUC15	mass spectrometry	bone	Prieto-Bonete et al. 2018 [25]
myoglobin		western blot	heart, skeletal muscle	Li et al. 2016 [27]
myosin heavy chain 2	MYH2	mass spectrometry	bone	Procopio et al. 2018 [59]
myosin heavy chain 6	MYH6	mass spectrometry	bone	Procopio et al. 2018 [59]
myristoylated alanine-rich C-kinase substrate	MARCKS	western blot	lung, skeletal muscle	Kang et al. 2003 [48]
myristoylated alanine-rich C-kinase substrate	MARCKS	western blot	lung, skeletal muscle	Poloz et al. 2009 [24]
Na+/K+ ATPase		enzyme activity assay	brain, liver, skeletal muscle, kidney	da Fonseca et al. 2019 [60]
nebulin		western blot	skeletal muscle	Foditsch et al. 2016 [57]
nebulin		western blot	skeletal muscle	Pittner et al. 2016 [23]
nicotinamide adenine dinucleotide phosphat dehydrogenase (ubiquinone), Fe_S protein 3		2D-PAGE	heart	Kwak et al. 2017 [52]
nicotinamide adenine dinucleotide phosphat dehydrogenase (ubiquinone), flavoprotein 1, 51 kDa		2D-PAGE	heart	Kwak et al. 2017 [52]
p53		western blot	kidney	Lee et al. 2016 [50]
pappalysin-1	PAPPA	mass spectrometry	bone	Prieto-Bonete et al. 2018 [25]
parkinson disease protein 7		2D-PAGE	liver	Kwak et al. 2017 [52]
peroxidasin homolog	PXDN	mass spectrometry	bone	Prieto-Bonete et al. 2018 [25]
peroxisome proliferator-activated receptor gamma	PPAR-γ	western blot	kidney	Lee et al. 2016 [50]
phosphoethanolamine/phosphocholine phosphatase isoform 1	PHOSPO1	mass spectrometry	bone	Prieto-Bonete et al. 2018 [25]
prelamin-A/C	LMNA	mass spectrometry	bone	Prieto-Bonete et al. 2018 [25]
procollagen C-endopeptidase enhancer 1 precursor	PCOLCE	mass spectrometry	bone	Prieto-Bonete et al. 2018 [25]
protein disulphide-isomerase	PDIA1	mass spectrometry	bone	Procopio et al. 2018 [59]
protein jagged-2	JAG2	mass spectrometry	bone	Prieto-Bonete et al. 2018 [25]
protein phosphatase 2	PP2A	western blot	lung, skeletal muscle	Poloz et al. 2009 [24]
proteoglycan		histology	cartilage	Alibegović et al. 2019 [46]
receptor for advanced glycation end products	RAGE	immunohistochemistry, western blot	lung	Cho and Eom 2019 [54]
Sarco/endoplasmic reticulum Ca 2+-ATPase 1	SERCA 2	western blot	skeletal muscle	Foditsch et al. 2016 [57]
Sarco/endoplasmic reticulum Ca 2+-ATPase 1	SERCA 1	western blot	skeletal muscle	Foditsch et al. 2016 [57]
sarco/endoplasmic reticulum Ca 2+-ATPase 1	SERCA 1	western blot	skeletal muscle	Pittner et al. 2016 [23]
scavenger receptor cysteine-rich type 1 protein M160	CD163L1	mass spectrometry	bone	Prieto-Bonete et al. 2018 [25]
serum albumin precursor		2D-PAGE	liver	Kwak et al. 2017 [52]
serum transferrin	TRFE	mass spectrometry	bone	Procopio et al. 2018 [59]
somatostatin		immunohistochemistry	brain, pankreas	Wehner et al. 2006 [29]
stress-70 protein, mitochondrial precursor		2D-PAGE	liver	Kwak et al. 2017 [52]
sulfate transporter SLC26A2		mass spectrometry	bone	Prieto-Bonete et al. 2018 [25]
SUN domain-containing ossification	SUCO	mass spectrometry	bone	Prieto-Bonete et al. 2018 [25]
thyroglobulin		immunohistochemistry	thyroid gland	Ortmann et al. 2017 [43]
thyroglobulin		immunohistochemistry	thyroid gland	Wehner et al. 2000 [33]
titin		western blot	skeletal muscle	Foditsch et al. 2016 [57]
titin		western blot	skeletal muscle	Pittner et al. 2016 [23]
Tnnt2 protein	Cardiac troponin T	2D-PAGE	heart	Kwak et al. 2017 [52]
transcription factor MAF	MAF	mass spectrometry	bone	Prieto-Bonete et al. 2018 [25]
transcription factor SOX-6 SOX6		mass spectrometry	bone	Prieto-Bonete et al. 2018 [25]
transcriptional protein SWT1 WNT11		mass spectrometry	bone	Prieto-Bonete et al. 2018 [25]
transferrin		2D-PAGE	heart	Kwak et al. 2017 [52]
triosephosphat isomerase	TPIS	mass spectrometry	bone	Procopio et al. 2018 [59]
tropomyosin	TPM	western blot	skeletal muscle	Choi et al. 2019 [47]
tropomyosin	CH1	western blot	skeletal muscle	Foditsch et al. 2016 [57]
tropomyosin	TPM	western blot	skeletal muscle	Pittner et al. 2016 [23]
tropomyosin	TPM	western blot	skeletal muscle	Pittner et al. 2016 [40]
tropomyosin	TPM	western blot	skeletal muscle	Pittner et al. 2017 [41]
tropomyosin	TPM	western blot	skeletal muscle	Zissler et al. 2018 [51]
tropomyosin 1, alpha isoform d		2D-PAGE	liver	Kwak et al. 2017 [52]
troponin I		western blot	skeletal muscle	Li et al. 2016 [27]
tubulin		western blot	heart, lung	Li et al. 2016 [27]
tubulin, beta 6		2D-PAGE	liver	Kwak et al. 2017 [52]
vascular endothelial growth factor	VEGF	ELISA	brain	Thaik-Oo et al. 2002 [34]
vinculin	VCL	western blot	skeletal muscle	Choi et al. 2019 [47]
vinculin		western blot	skeletal muscle	Zissler et al. 2018 [51]
zinc finger CCHC domain-containing protein 12		mass spectrometry	bone	Prieto-Bonete et al. 2018 [25]
