# Supplementary material for: Postmortem Protein Degradation as a Tool to Estimate the PMI: A Systematic Review
Source: Diagnostics (Basel). 2020 Nov 26;10(12):1014. doi: 10.3390/diagnostics10121014 (PMC7760775; doi:10.3390/diagnostics10121014)
Supplement: Supplementary file 1 [file diagnostics-10-01014-s001.zip › Table S2.pdf]

Domain	Question	Wehner et al. 1999 [31]	Wehner et al. 2000 [33]	Wehner et al. 2001 [32]	Wehner et al. 2001 [30]	Thaik-Oo et al. 2002 [34]	Kang et al. 2003 [48]	Sabucedo and Furton 2003 [35]	Wehner et al. 2006 [29]	Poloz and O'Day 2009 [24]	Boaks et al. 2014 [55]	Bolton et al. 2015 [56]	Kumar et al 2015 [37]	Kumar et al. 2015 [36]	Kumar et al. 2015 [38]	Abo El-Noor et al. 2016 [49]	Lee et al. 2016 [50]	Kumar et al. 2016 [39]	Foditsch et al. 2016 [57]	Pittner et al. 2016 [23]	Pittner et al. 2016 [40]	Kwak et al. 2017 [52]	Li et al. 2017 [44]	Li et al. 2017 [53]	Ortmann et al. 2017 [43]	Pérez-Martínez et al. 2017 [42]	Pittner et al. 2017 [41]	Jellinghaus et al. 2018 [58]	Prieto-Bonete et al. 2018 [25]	Procopio et al. 2018 [59]	Zissler et al. 2018 [51]	Alibegovic et al. 2019 [46]	Cho and Eom 2019 [54]	Choi et al. 2019 [47]	da Fonseca et al. 2019 [60]	Jellinghaus et al. 2019 [45]
Bias arising from study design	1.1 Was the sample size chosen adequately (to appropriately address the research hypothesis)? (Y/PY/N/PN/NI)	Y	Y	Y	Y	PY	PY	PN	Y	PY	PN	PN	PN	N	PN	Y	Y/N[1]	N	N	PY	Y	PN	PY	N	Y	Y	N	Y	Y	N	PY	N	Y	Y/N[4]	Y	Y
	1.2.1 If Y/PY to 1.1 Were appropriate control samples used that serve as references/comparative values within the outcome analysis domain? (Y/PY/N/PN/NI)	Y	Y	Y	Y	Y	Y		Y	Y						Y	Y/N[1]			Y	Y		Y		Y	Y		Y	Y		Y		Y	Y/N[4]	Y	Y
	1.2.2 If Y/PY to 1.2.1 Was there any effort to reduce risk of bias in selecting an appropriate population fitting into the context of the study? (Y/PY/N/PN/NI)	Y	Y	Y	Y	N	Y		Y	PY						Y	Y/N[1]			Y	Y		Y		Y	N		N	N		Y		Y	Y/N[4]	Y	N
	**Risk of bias judgement**	low	low	low	low	moderate	low	high	low	low	high	high	high	high	high	low	low/high	high	high	low	low	high	low	high	low	moderate	high	moderate	moderate	high	low	high	low	low/high	low	moderate
Bias due to imprecise reporting	2.1 Was the study design and outcome reported consistently and unambigously?	Y	Y	Y	Y	Y	Y	N	Y	Y	Y	Y	N	N	N	Y	Y	N	Y	Y	Y	Y	Y	Y	Y	Y	Y	Y	Y	Y	Y	Y	Y	Y	Y	N
	2.2 Was the sampling (procedure and site) reported comprehensibly? (Y/N)	Y	Y	Y	Y	Y	N	N	Y	N	Y	Y	N	N	N	Y	Y	N	Y	Y	Y	Y	Y	Y	Y	N	Y	Y	Y	Y	Y	Y	Y	Y	Y	N
	2.3 Were influencing factors and/or environmental conditions reported that possibly influence the outcome domain? (Y/N)	Y	Y	Y	Y	N	Y	Y	Y	Y	N	Y	Y	N	N	Y	Y	Y	Y	Y	Y	Y	Y	Y	Y	Y	Y	Y	Y	N	Y	Y	Y	Y	Y	N
	2.4 Were methodical steps reported comprehensively and traceable from sample preparation to data acquisition? (Y/N)	N	N	N	N	Y	Y	N	N	Y	Y	Y	Y	Y	Y	Y	Y	Y	Y	Y	Y	Y	Y	Y	N	Y	Y	Y	Y	Y	Y	Y	Y	Y	Y	Y
	2.5 Were details reported regarding the measurement procedure and data analysis that are essential for the outcome domain (endpoint of interest)? (Y/N)	N	N	N	N	N	Y	N	N	Y	Y	N	N	N	N	Y	Y/N[2]	N	N	N	Y	N	Y	Y	N	Y	Y	Y	Y	Y	Y	Y	Y/N[3]	Y	Y	Y
	**Risk of bias judgement**	high	high	high	high	high	moderate	high	high	moderate	moderate	moderate	high	high	high	low	low/moderate	moderate	moderate	moderate	low	moderate	low	low	high	moderate	low	low	low	moderate	low	low	moderate/low	low	low	high
Bias due to missing outcome data	3.1. Were outcome data available for all, or nearly all, samples used? (Y/PY/N/PN/NI)	Y	Y	Y	Y	N	Y	PY	PN	Y	Y	PY	PY	PY	N	Y	Y/N	N	PY	Y	Y	Y	PY	Y	Y	Y	Y	Y	Y	Y	Y	Y	PY	Y	Y	PY
	3.2.1. If N/PN/NI to 3.1 Are the proportions of missing outcome data similar across study groups? (Y/PY/N/PN/NI)					PN			N						NI			NI																		
	3.2.2. If N/PN/NI to 3.1 Are the reasons for (partially) missing outcome data reported? (Y/N)					N			N						N			N																		
	3.2.3. If N/PN/NI to 3.1 Is there evidence that results were robust to the presence of missing outcome data? (Y/NI/N)					N			NI						N			N																		
	**Risk of bias judgement**	low	low	low	low	high	low	low	high	low	low	low	low	low	high	low	low	high	low	low	low	low	low	low	low	low	low	low	low	low	low	low	low	low	low	low
Bias in measurement of the outcome	4.1. Were outcome assessors aware of the study group/time point/individual sample data? (Y/PY/NI/N/PN)	NI	NI	NI	NI	NI	NI	NI	NI	NI	NI	NI	NI	NI	NI	NI	NI	NI	NI	NI	NI	ni	NI	NI	NI	NI	NI	NI	NI	NI	NI	PN	NI	NI	NI	N
	4.2. If Y/PY/NI to 4.1 Was the assessment of the outcome likely to be influenced by knowledge of the study group/time point/individual sample data? (Y/PY/N/PN)	PY	PY	PY	PY	PN	PN	PN	PY	PN	PN	PY	PN	PN	PN	PN	PN	PN	PY	PY	PN	PN	PN	PN	PY	PN	PN	PN	PN	PN	PN	PN	PN	PN	PN	
	**Risk of bias judgement**	moderate	moderate	moderate	moderate	low	low	low	moderate	low	low	moderate	low	low	low	low	low	low	moderate	moderate	low	low	low	low	moderate	low	low	low	low	low	low	low	low	low	low	low
Bias in selection of the reported result	5.1 Are the reported outcome data likely to have been selected from multiple outcome measurements (methods, time points, conditions, etc.)? (Y/PY/N/PN/NI)	N	N	N	N	PN	N	PN	N	N	N	N	PN	PN	PN	N	PN	PY	PN	N	N	N	N	N	N	N	N	N	N	N	N	N	N	N	PN	PN
	5.2 Are the reported outcome data likely to have been selected from multiple analyses of the data (values, percentages, ratios)? (Y/PY/N/PN/NI)	N	N	N	N	N	N	N	N	N	N	N	N	N	N	N	N	N	N	N	N	N	N	N	N	PY	N	N	PY	N	N	N	N	N	N	N
	**Risk of bias judgement**	low	low	low	low	low	low	low	low	low	low	low	low	low	low	low	low	moderate	low	low	low	low	low	low	low	moderate	low	low	moderate	low	low	low	low	low	low	low
Bias arising from multiple use of data	6.1 Is there evidence that the same **individuals** were used in other included studies, published earlier? (Y/PY/N/PN)	N	Y	Y	Y	N	N	N	PY	N	N	N	N	N	PY	N	N	N	N	N	N	N	N	N	N	PY	PN	N	PY	N	N	N	N	PN	N	N
	6.2.1 If Y/PY to 6.1 Is there evidence that the same **sample** material was used in other included studies, published earlier? (Y/PY/N/PN)		N	Y	Y				PY						PY											PY			PY					PN		
	6.2.2 If Y/PY to 6.2.1 Is the multiple use of samples likley to affect meta-analysis? (Y/PY/N/PN)			N	N				N						Y											PN			PN					PN		
	6.3 If Y/PY to 6.2.1 Is there evidence that the same **data** were used in other included studies, published earlier? (Y/PY/N/PN)		N	N	N				N						PY											N			N					N		
	**Risk of bias judgement**	low	moderate	moderate	moderate	low	low	low	moderate	low	low	low	low	low	high	low	low	low	low	low	low	low	low	low	low	moderate	low	low	moderate	low	low	low	low	low	low	low
Overall bias	**Risk of bias judgement**	high	high	high	high	high	moderate	high	high	moderate	high	high	high	high	high	low	low/moderate/high	high	high	moderate	low	high	low	high	high	moderate	high	moderate	moderate	moderate	low	high	moderate/low	low/high	low	high

[1] Y for WB and IHC, N for LFA
[2] Y for WB analysis, N for IHC
[3] Y for WB analysis, N for IHC
[4] Y for WB analysis in rats, N for MS analysis in animals and human
